# The Effect of Time-Restricted Eating on Cardiometabolic Risk Factors: A Systematic Review and Meta-Analysis

**DOI:** 10.3390/nu16213700

**Published:** 2024-10-30

**Authors:** Krystalia Panagiotou, Garyfallia Stefanou, Georgia Kourlaba, Dimitrios Athanasopoulos, Penio Kassari, Evangelia Charmandari

**Affiliations:** 1 Master of Sciences (MSc) Program “General Pediatrics and Pediatric Subspecialties: Clinical Practice and Research”, National and Kapodistrian University of Athens Medical School, 11527 Athens, Greece; krystalia_pn@hotmail.com (K.P.); dimitrios.g.athanasopoulos@gmail.com (D.A.); peniokassari@gmail.com (P.K.); 2 ECONCARE—Health Research & Consulting, 11528 Athens, Greece; g_stefanou@hotmail.com; 3 Nursing Department, University of the Peloponnese, 22131 Tripoli, Greece; 4 Center for the Prevention and Management of Overweight and Obesity, Division of Clinical and Translational Research in Endocrinology, First Department of Pediatrics, National and Kapodistrian University of Athens Medical School, ‘Aghia Sophia’ Children’s Hospital, 11527 Athens, Greece; 5 Division of Endocrinology and Metabolism, Center of Clinical, Experimental Surgery and Translational Research, Biomedical Research Foundation of the Academy of Athens, 11527 Athens, Greece

**Keywords:** time-restricted eating, circadian rhythm, obesity, meta-analysis

## Abstract

**Background/Objectives:** Endogenous metabolic pathways periodically adjust with fluctuations in day and night, a biological process known as circadian rhythm. Time-restricted eating (TRE) aligns the time of food intake with the circadian rhythm. This study aims to investigate the effects of TRE on body weight, body composition and cardiometabolic risk factors. **Methods:** We reviewed articles from PubMed and Cochrane libraries for clinical trials that compare TRE with regular diet without calorie restriction. We conducted a meta-analysis of 26 studies. **Results:** Participants who followed TRE demonstrated reduction in body weight [mean-MD: −1.622 kg, (95% confidence interval (CI −2.302 to −0.941)], body mass index (BMI) [MD: −0.919 kg/m^2^ (95% CI: −1.189 to −0.650)], waist circumference [MD: −2.015 cm (95% CI: −3.212 to −0.819] and whole-body fat mass (WBFM) [MD: −0.662 kg (95% CI: −0.795 to −0.530)]. Improvements in cardiometabolic risk factors such as a decrease in insulin concentrations [MD: −0.458 mIU/L, (95% CI: −0.843 to −0.073)], total cholesterol [MD: −2.889 mg/dL (95% CI: −5.447 to −0.330) and LDL concentrations [MD: −2.717 mg/dL (95% CI: −4.412 to −1.021)] were observed. **Conclusions:** TRE is beneficial for weight loss and improvements in cardiometabolic risk factors. Further large-scale clinical trials are needed to confirm these findings.

## 1. Introduction

The circadian rhythm is an intrinsic self-sustained biological process based on the light–dark cycles within the period of one day, as the Earth rotates around itself [1] Thus, the circadian rhythm periodically coordinates and regulates a variety of metabolic pathways of an organism according to environmental changes and external cues [2].

In the last few decades, it has been proven that the majority of eukaryotes and many procaryotes have evolved in order to follow the circadian rhythm, which generates a number of endogenous hierarchic oscillations [3]. This multi-oscillatory system consists of a master circadian clock in the central nervous system (CNS) and multiple subordinate clocks in other brain regions and in most peripherals tissues [4].

**Figure 1:**
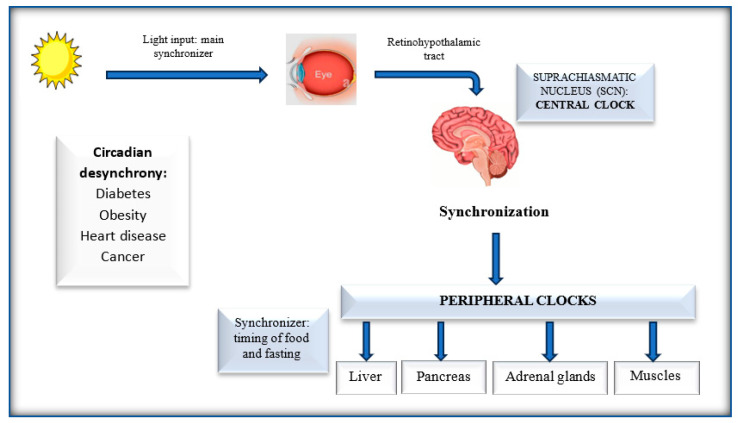
Circadian clocks.

In mammals, the suprachiasmatic nucleus (SCN) in the hypothalamus is the central circadian pacemaker, situated directly above the optic chiasm and comprised of 20.000 neurons [5]. The SCN receives photic stimuli from the retina via the retino-hypothalamic tract [6]. Therefore, light is the main synchronizer for the SCN, whereas the timing of food and fasting affect the periodic function of the peripheral tissues [7]. The peripheral clocks are located in other brain regions (hypothalamic nuclei, forebrain, olfactory bulb, and pineal gland) and in other non-neuronal tissues (liver, kidney, muscle, adipose tissue, blood cells, adrenal glands, stomach, intestine, and pancreas) [6,7] (Figure 1).

At the molecular level, the rhythmic function of the central clock and the peripheral clocks is based on complex transcriptional–translational feedback loops, including “clock genes” and their protein products [8]. Moreover, the synchronization between the CNS and the peripheral tissues involves both direct neuronal and indirect hormonal signals [5]. The rhythmic circulation of glucocorticoids, such as cortisol, is the most crucial internal stimulus because of the abundance of their receptors in most of the tissues. The other important part of this system is melatonin, which also has feedback effects on the SCN. The other form of interaction consists of sympathetic and parasympathetic branches of the autonomous nervous system [9].

**Figure 2:**
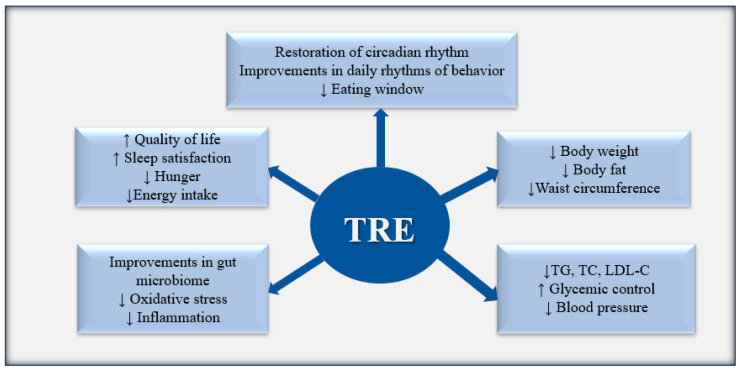
Overall positive effects of TRE (time-restricted eating). ↑ Indicates increase; ↓ Indicates decrease.

Several studies have demonstrated that prolonged circadian desynchrony has detrimental metabolic consequences for human health, such as insulin resistance, dyslipidemia, and hyperglycemia [10]. Therefore, given that food timing is the main internal cue for the peripheral organs, dietary changes can have many health benefits, restoring circadian and metabolic homeostasis [11] (Figure 2).

Time-restricted eating (TRE) refers to a dietary pattern in which daily caloric intake is restricted to a time window of approximately 4–12 h, which induces a fasting window of 12–20 h per day [12]. Controlled animal and human studies showed that TRE prevents or attenuates the severity of several metabolic diseases, including obesity, glucose intolerance, hepatic steatosis, dyslipidemia, and age-related decline in cardiac function [13,14,15]. TRE does not require daily caloric restriction, as the limitation of the eating duration can reduce the total energy intake by approximately ~350–500 kcal/day [16]. Numerous clinical trials have been conducted recently in order to investigate the effects of TRE in comparison with other nutritional plans and have included participants with or without metabolic disorders. Other researchers have already investigated the effect of TRE on several outcomes relating to body composition and metabolic parameters, acknowledging its positive effects on several outcomes [17,18]. In this study, we intend to investigate the beneficial effects of TRE on body composition and metabolic parameters, such as body weight, glucose metabolism, blood pressure, and lipid profile. We aim to provide an up-to-date systematic review and meta-analysis of the latest clinical trials (up to 2023) that largely study the 16:8 dietary model, without calorie restriction, compared to no dietary intervention.

## 2. Materials and Methods

### 2.1. Search Strategy

Two investigators conducted research in two citation databases, PubMed and Cochrane Library, using the same keywords in English. The keywords used were «time-restricted eating», «time-restricted diet», «time-restricted feeding», «time-restricted meal», «time-restricted fasting», «intermittent fasting», «periodic fasting», «circadian fasting», «time-limited eating», «chrono-nutrition», and «temporal eating». For parameters conferring cardiometabolic risk, the keyworks used were «weight», «blood pressure», «hypertension», «insulin», «glucose», «total cholesterol», «triglycerides», «low-density lipoprotein cholesterol» (LDL-C), «high-density lipoprotein cholesterol» (HDL-C), «waist circumference», «waist-to-hip ratio», «waist-to-height ratio», «insulin resistance», «prediabetes», and «insulin resistance index». In addition, we conducted Google searches of the gray literature.

### 2.2. Study Selection

The same two investigators excluded studies by title, abstract and full text. The inclusion criteria were (1) population, meaning all age groups, including children and adolescents; (2) intervention, meaning a daily fasting period of 12–20 h; (3) study design, referring to RCTs or non-RCTs using TRE; (4) outcomes, meaning data on changes in at least one of the factors of weight, BMI, blood pressure, insulin, glucose, total cholesterol, triglycerides, low-density lipoprotein cholesterol (LDL-C), high-density lipoprotein cholesterol (HDL-C), waist circumference, hip circumference, and waist-to-hip ratio; and (5) comparators, referring to a control group in randomized controlled trials (RCTs) or non-randomized controlled trials or subjects before TRE intervention in studies with a one group pretest–posttest design.

**Figure 3:**
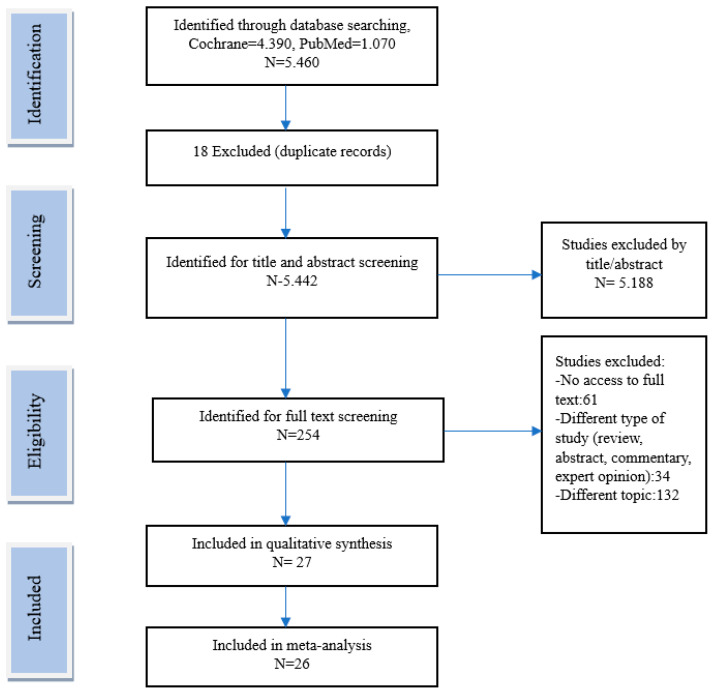
Search strategy.

Exclusion criteria were as follows: (1) articles on animal studies or in vivo experiments; (2) studies including participants with acute or chronic diseases, such as gastrointestinal diseases or cancer, that could affect the outcomes; (3) studies with insufficient information on the TRE regimen or studies including intermittent or periodic fasting or energy restriction; (4) studies with abstracts only, and non-original articles, including expert opinions or reviews; and (5) studies on religious fasting, including Ramadan fasting. The database search yielded a total of 5460 records, with 4390 records retrieved from the Cochrane database and 1070 records from PubMed. The initial search took place on April 2022. In order to update the results of the research, a second database search took place on July 2024. After the selection process, 27 studies were included for qualitative synthesis and 26 of those studies found to be suitable for inclusion in the meta-analysis (Figure 3) (Table 1).

### 2.3. Data Extraction

Two investigators independently created Microsoft Excel tables in order to extract data from the studies, and they cross-checked their results. The same variables (first author, publication year, country, study design, characteristics of the participants, number of study participants, duration of the study, mean age, sex, anthropometric data, body composition, blood pressure, glucose, insulin and HbA1C concentration, and lipid profile) were used.

### 2.4. Quality Assessment Analysis

We used the “Revised Cochrane risk-of-bias tool for randomized trials (ROB-2.0)” tool to assess the quality of RCTs and “Risk of Bias in Non-randomized Studies of Interventions (ROBINS-I)” tool to assess non-randomized clinical trials (non-RCTs). We assessed the risk of bias for RCTs by examining the randomization process, deviations from intended interventions, missing outcome data, measurement of outcomes, and selection of the reported results. We assessed the risk of bias for non-RCTs by examining the classification of interventions, deviations from intended interventions, missing outcome data, measurement of outcomes, selection of the reported result, and bias due to confounding and selection of participants. The risk associated with each domain was classified as low, some concerns, or high.

### 2.5. Data Analyses and Statistical Methods

For all continuous variables, the mean and standard deviation or 95% confidence interval (CI) of the differences observed from study entry to study end in each group were recorded, depending on the availability of information. A meta-analysis of the absolute mean difference of the mean values (unstandardized mean differences) of the outcome values between the intervention group and the control group was performed with fixed and random-effects models. For the fixed-effects model, the inverse–variance method was used, while for the random-effects model, the DerSimonian and Laird method was used. Heterogeneity between studies was assessed using the Q statistic, followed by calculating the *I*^2^ statistic to quantify the percentage of variability attributable to heterogeneity. For outcomes where heterogeneity was statistically significant, meta-regression analyses were conducted to explore potential sources of variability. The factors considered in the meta-regression included geographical region (USA, European countries, Australia, China, Brazil), study duration (in weeks), health status (healthy, metabolic syndrome), percentage of female participants, age (pooled mean), BMI (pooled mean), study design (randomized, non-randomized, crossover), fasting duration (hours), and risk of bias (low, moderate, serious). To assess the potential impact of bias on our findings, we conducted subgroup analyses. Specifically, we performed separate analyses for studies with low risk of bias compared to those with some concerns or serious risk of bias in case of high heterogeneity. Publication error was checked using funnel plots. The statistical significance level was set at 5%. All analyses were conducted with the statistical program STATA 17.0.

## 3. Results

### 3.1. Study Characteristics

The 27 studies included in the qualitative synthesis and meta-analysis were published between 2007 and 2023. The total number of participants included across all studies was approximately 1.197, with study sizes ranging from 8 to 174 participants. The majority of the studies were RCTs [19,20,21,22,23,24,25,26,27,28,29,30,31,32,33,34,35]. In addition, 3 studies were non-RCTs [31,36,37] and 7 were randomized crossover studies [38,39,40,41,42,43,44]. One study was a single-arm trial and was not included in the meta-analysis [45]. There were 13 studies with healthy individuals [23,26,27,28,29,30,31,32,34,37,38,42,43,44], while the rest of them included subjects with metabolic abnormalities, such as overweight or obesity, prediabetes or metabolic syndrome [19,20,21,22,24,25,33,35,36,39,40,41,45]. These studies encompassed a range of interventions related to time-restricted eating. In 18 studies, the participants followed the 16:8 schedule (16 h of fasting) [19,22,23,24,25,26,27,28,29,31,32,33,35,36,38,40,44]. In the remaining 9 studies, the participants followed other dietary plans, such as 12:12 [20,37], 10:14 [21,34], 14:10 [45], 18:6 [39,41], 20:4 [30,43], and 22:2 [42]. The duration of the intervention varied from 4 days to 12 months.

### 3.2. Quality Assessment Results

**Figure 4:**
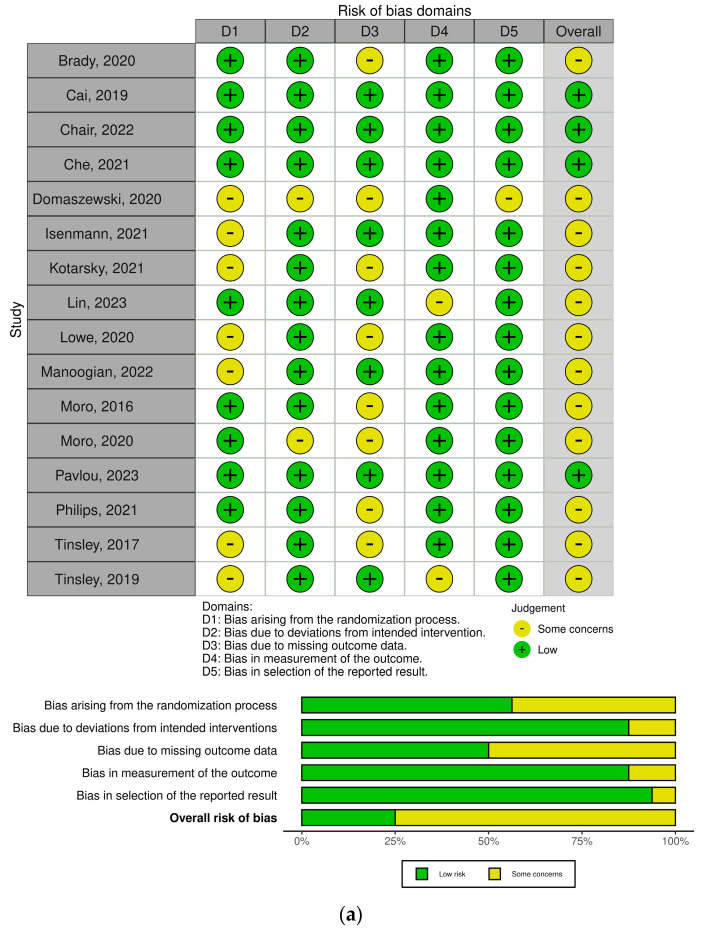

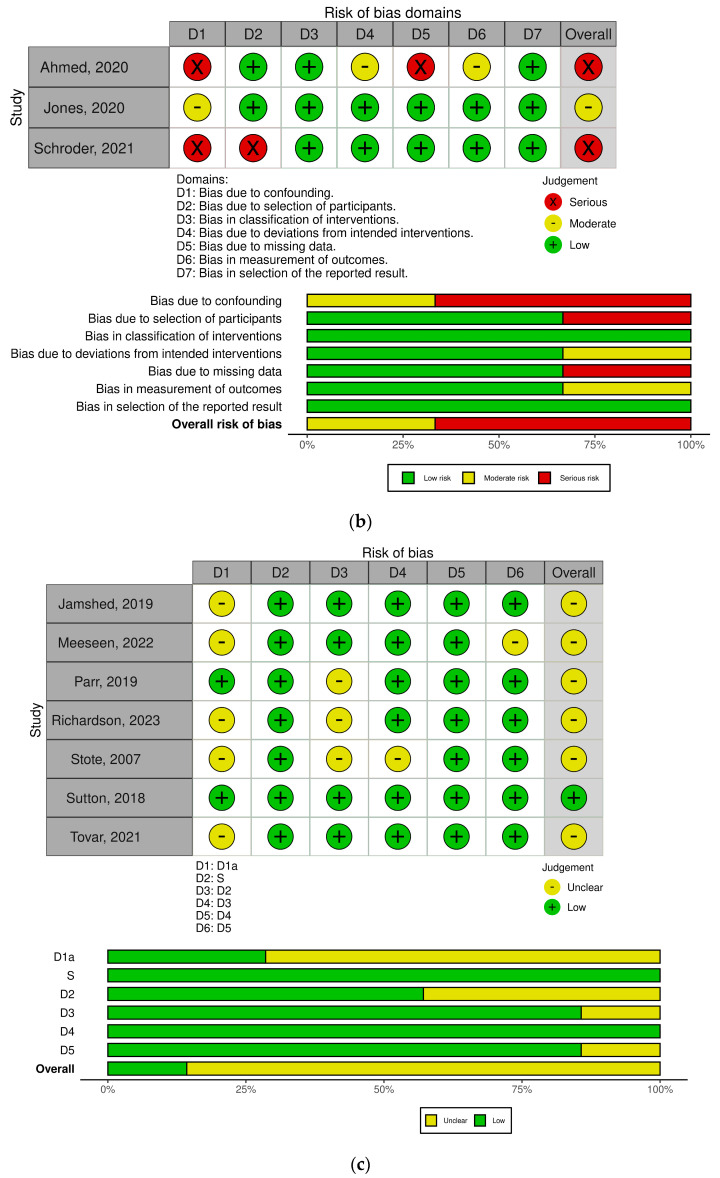
Risk-of-bias assessment in the studies included in the meta-analysis. (**a**): Randomized control trials, (**b**): non-randomized control trials, (**c**) crossover [19,20,21,22,23,24,25,26,27,28,29,30,31,32,33,34,35,36,37,38,39,40,41,42,43,44].

The risk of bias for randomized clinical studies was assessed as moderate for the majority of the studies, since 12 of the 15 studies were deemed to have “Some concerns”, mainly for the randomization performed and for the set of missing values observed at the end of each study [19,20,23,24,26,27,28,29,30,32,33,34]. The risk of bias for crossover clinical studies was also assessed as moderate for the majority of studies, as six of the seven studies were deemed to have “Some concerns”, i.e., there were some concerns about the methods applied, and these were mainly related to randomization methods [38,40,41,42,43,44]. The risk of bias for non-randomized clinical studies was assessed as serious, since two of the three studies had serious errors, which were mainly due to failure to control for confounding factors [36,37] (Figure 4).

### 3.3. Effect of TRE on Body Mass Index (BMI) and Weight

**Figure 5:**
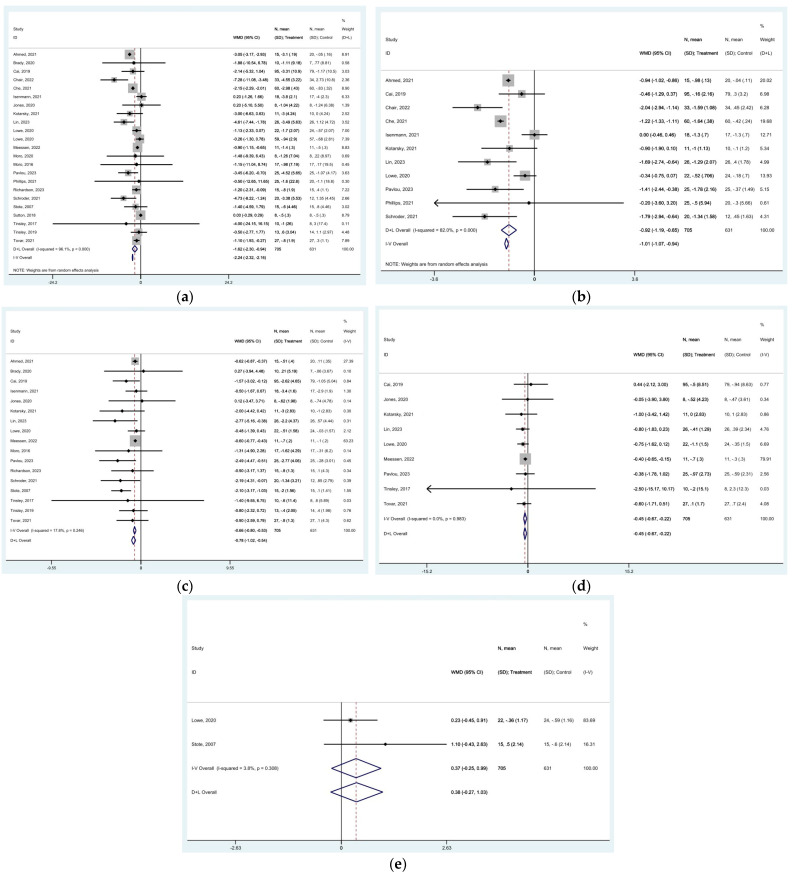
Forest plots summarizing the effect of TRE on (**a**) body weight, (**b**) BMI, (**c**) whole-body fat mass, (**d**) lean mass, and (**e**) total body water. Squares 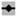 represent effect estimates for each study, with size proportional to study weight. Horizontal lines indicate 95% confidence intervals (CI). The diamond 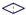 shows the pooled effect, with its width representing the 95% CI. The vertical line 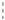 through the diamond represents the overall effect estimate. The central vertical line 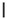 at zero marks no effect; confidence intervals crossing this line indicate non-significant results. “D+L” and “IV” indicate different pooling methods (random-effects and fixed-effects, respectively). *I*^2^ and *p*-value indicate heterogeneity across studies [19,20,21,22,23,24,25,26,28,29,30,31,32,33,35,36,37,38,39,42,43,44].

The meta-analysis showed a significant weight reduction using the random-effects model with mean difference (MD) of −1.622 kg (95% CI: −2.302 to −0.941, *p* < 0.0001), with significant heterogeneity among studies (*I*^2^ = 96.1%, *p* < 0.0001) (Figure 5a). The funnel plot was symmetric (Supplemental Figure S1a). BMI also decreased after the TRE intervention [MD of −0.919 kg/m^2^ (95% CI: −1.189 to −0.650, *p* < 0.0001), with high heterogeneity among studies (*I*^2^ = 82%, *p* < 0.0001)] (Figure 5b). The funnel plot was not symmetric due to publication bias (Supplemental Figure S1b).

### 3.4. Effect of TRE on Whole-Body Fat Mass (WBFM), Lean Mass (LM), and Total Body Water (TBW)

The meta-analysis indicated a significant reduction in whole-body fat mass (WBFM) after the TRE intervention [MD of −0.662 kg (95% CI: −0.795 to −0.530, *p* < 0.0001), with low heterogeneity among studies (*I*^2^ = 17.8%, *p* = 0.246)] (Figure 5c). The funnel plot was symmetric (Supplemental Figure S1c). In addition, there was a slight decrease in lean mass (LM) [MD of −0.448 kg (95% CI: −0.672 to −0.224, *p* < 0.0001), with no heterogeneity among studies (*I*^2^ = 0.0%, *p* = 0.983)] (Figure 5d). The funnel plot was symmetric (Supplemental Figure S1d). Total body water demonstrated a slight non-significant increase after TRE [MD of 0.372 kg (95% CI: −0.246 to 0.990, *p* = 0.238), with low heterogeneity among studies (*I*^2^ = 3.8%, *p* = 0.308)] (Figure 5e). The funnel plot was not symmetric (Supplemental Figure S1e).

### 3.5. Effect of TRE on Body Measurements

**Figure 6:**
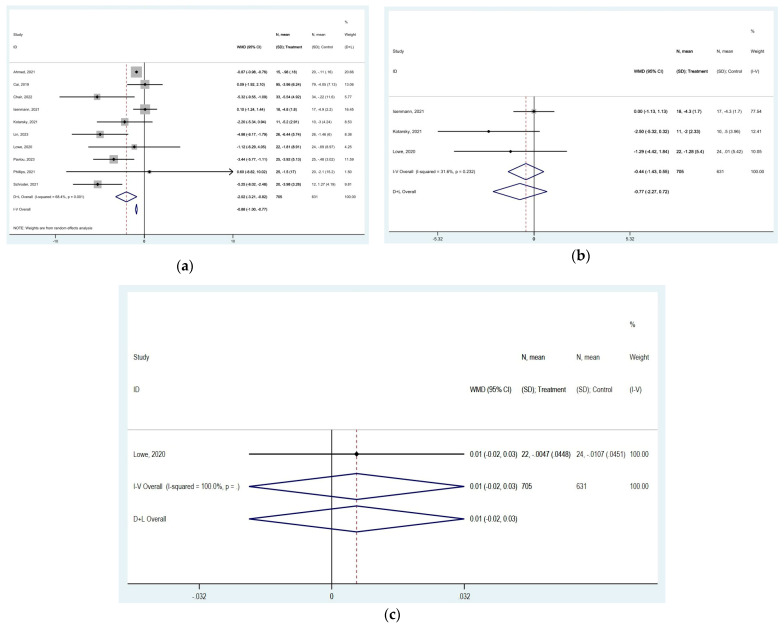
Forest plots summarizing the effect of TRE on (**a**) waist circumference, (**b**) hip circumference, and (**c**) waist-to-hip ratio. Squares 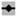 represent effect estimates for each study, with size proportional to study weight. Horizontal lines indicate 95% confidence intervals (CI). The diamond 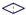 shows the pooled effect, with its width representing the 95% CI. The vertical line 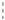 through the diamond represents the overall effect estimate. The central vertical line 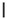 at zero marks no effect; confidence intervals crossing this line indicate non-significant results. “D+L” and “IV” indicate different pooling methods (random-effects and fixed-effects, respectively). *I*^2^ and *p*-value indicate heterogeneity across studies [19,20,22,24,25,26,33,35,36,37].

Waist circumference showed a significant reduction after TRE [MD of −2.015 cm (95% CI: −3.212 to −0.819, *p* = 0.001), with high heterogeneity among studies (*I*^2^ = 68.4%, *p* = 0.001)] (Figure 6a). The funnel plot was not symmetric due to publication bias (Supplemental Figure S2a). Hip circumference showed no significant change [MD of −0.440 cm (95% CI: −1.432 to 0.552, *p* = 0.385), with low heterogeneity among studies (*I*^2^ = 31.6%, *p* = 0.232)] (Figure 6b). The funnel plot was not symmetric due to publication bias (Supplemental Figure S2b). Waist-to-hip ratio was measured only in one study [17] and showed a slight no significant increase after TRE [MD of 0.006 cm (95% CI: −0.020 to 0.032, *p* = 0.651)] (Figure 6c). The funnel plot showed no results (Supplemental Figure S2c).

### 3.6. Effect of TRE on Blood Pressure

**Figure 7:**
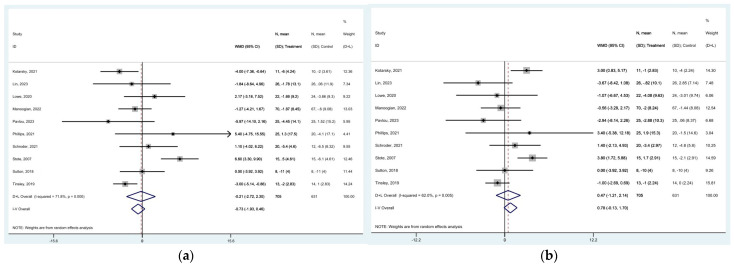
Forest plots summarizing the effect of TRE on (**a**) SBP and (**b**) DBP. Squares 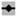 represent effect estimates for each study, with size proportional to study weight. Horizontal lines indicate 95% confidence intervals (CI). The diamond 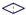 shows the pooled effect, with its width representing the 95% CI. The vertical line 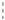 through the diamond represents the overall effect estimate. The central vertical line 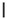 at zero marks no effect; confidence intervals crossing this line indicate non-significant results. “D+L” and “IV” indicate different pooling methods (random-effects and fixed-effects, respectively). *I*^2^ and *p*-value indicate heterogeneity across studies [19,20,24,28,33,34,35,36,39,43].

The systolic blood pressure showed no significant change after TRE intervention [MD of −0.212 mmHg (95% CI: −2.721 to 2.298, *p* = 0.869), with high heterogeneity among studies (*I*^2^ = 71.8%, *p* < 0.0001)] (Figure 7a). Similarly, the diastolic blood pressure demonstrated no significant change [MD of 0.466 mmHg (95% CI: −1.207 to 2.140, *p* = 0.585), with high heterogeneity among studies (*I*^2^ = 62%, *p* = 0.005)] (Figure 7b). The funnel plots for both systolic and diastolic blood pressure were asymmetric due to publication bias (Supplemental Figure S3a,b).

### 3.7. Effect of TRE on Metabolic Parameters

**Figure 8:**
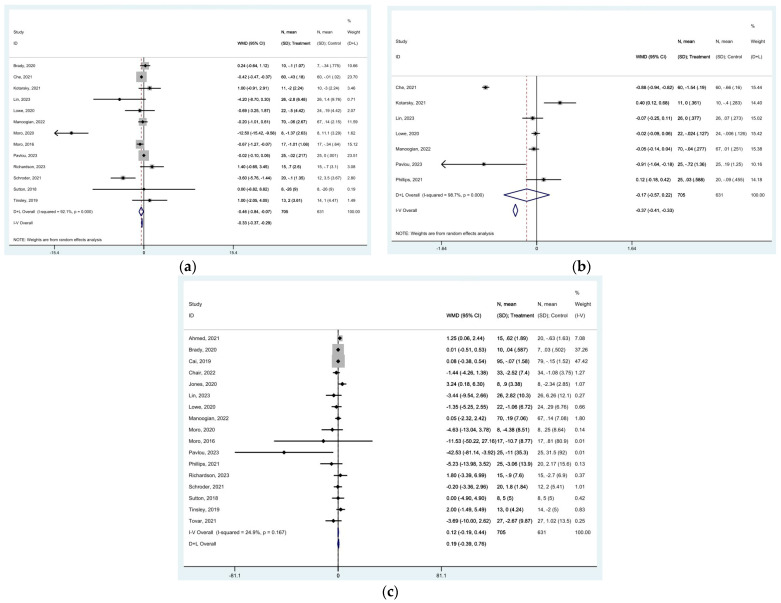
Forest plots summarizing the effect of TRE on (**a**) insulin concentrations, (**b**) HbA1C concentrations, and (**c**) glucose concentrations. Squares 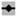 represent effect estimates for each study, with size proportional to study weight. Horizontal lines indicate 95% confidence intervals (CI). The diamond 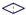 shows the pooled effect, with its width representing the 95% CI. The vertical line 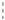 through the diamond represents the overall effect estimate. The central vertical line 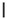 at zero marks no effect; confidence intervals crossing this line indicate non-significant results. “D+L” and “IV” indicate different pooling methods (random-effects and fixed-effects, respectively). *I*^2^ and *p*-value indicate heterogeneity across studies [19,20,21,22,23,24,25,28,29,31,32,33,34,35,36,37,38,39,44].

TRE resulted in reduced insulin concentrations [MD of −0.458 mIU/L (95% CI: −0.843 to −0.073, *p* = 0.020), with high heterogeneity among studies (*I*^2^ = 92.1%, *p* < 0.0001)] (Figure 8a). The funnel plot was not symmetric due to publication bias (Supplemental Figure S4a). Furthermore, TRE lowered HbA1C concentrations [MD of −0.175% (95% CI: −0.569 to 0.219, *p* = 0.385), with high heterogeneity among studies (*I*^2^ = 98.7%, *p* < 0.0001)] (Figure 8b). The funnel plot was not symmetric due to publication bias (Supplemental Figure S4b). However, glucose concentrations showed a slight non-significant increase [MD of 0.124 mg/dL (95% CI: −0.193 to 0.442, *p* = 0.444), with low heterogeneity among studies (*I*^2^ = 24.9%, *p* = 0.167)] (Figure 8c). The funnel plot was symmetric due to a lack of significant publication bias (Supplemental Figure S4c).

**Figure 9:**
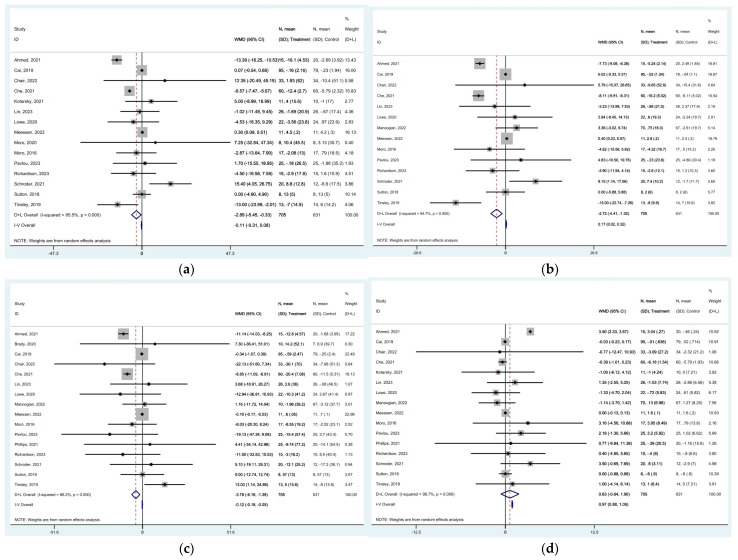
Forest plots summarizing the effect of TRE on (**a**) total cholesterol concentrations, (**b**) LDL concentrations, (**c**) triglyceride concentrations, and (**d**) HDL concentrations. Squares 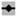 represent effect estimates for each study, with size proportional to study weight. Horizontal lines indicate 95% confidence intervals (CI). The diamond 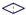 shows the pooled effect, with its width representing the 95% CI. The vertical line 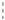 through the diamond represents the overall effect estimate. The central vertical line 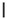 at zero marks no effect; confidence intervals crossing this line indicate non-significant results. “D+L” and “IV” indicate different pooling methods (random-effects and fixed-effects, respectively). *I*^2^ and *p*-value indicate heterogeneity across studies [19,20,21,22,23,24,25,28,29,32,33,34,35,36,37,39,42,44].

As far as the lipid profile is concerned, total cholesterol concentrations decreased following TRE [MD of −2.889 mg/dL (95% CI: −5.447 to −0.330, *p* = 0.027), with high heterogeneity among studies (*I*^2^ = 95.5%, *p* < 0.0001)] (Figure 9a). TRE also resulted in decreased LDL concentrations [MD of −2.717 mg/dL (95% CI: −4.412 to −1.021, *p* = 0.002), with high heterogeneity among studies (*I*^2^ = 94.7% *p* < 0.0001)] (Figure 9b). Significant changes were reported in triglycerides concentrations [MD of −3.782 mg/dL (95% CI: −6.180 to 1.384, *p* = 0.002), with high heterogeneity among studies (*I*^2^ = 88.2%, *p* < 0.0001)] (Figure 9c), while HDL showed no significant increase following TRE [MD of 0.632 mg/dL (95% CI: −0.636 to 1.899, *p* = 0.329), with high heterogeneity among studies (*I*^2^ = 98.7%, *p* < 0.0001) (Figure 9d). The funnel plots for total cholesterol, LDL, HDL, and triglyceride concentrations were not symmetric (Supplemental Figures S5a–S5d). A summary of the results is presented in Table 2. 

### 3.8. Evaluation of Heterogeneity

For outcomes where heterogeneity was statistically significant, meta-regression analyses were conducted to explore potential sources of variability (Table 3). The results showed that the country where the studies were conducted significantly explains the heterogeneity in insulin and HbA1C levels. The adjusted R^2^ values are very high (92.9% and 94.2%), indicating that most of the between-study variation in insulin and HbA1C outcomes can be explained by differences between countries. Specific comparisons (China vs. USA, Brazil vs. USA, etc.) showed significant effects for insulin and HbA1C levels. For example, China vs. USA showed a reduction in insulin levels (−4.92, 95% CI: −6.96, −2.87), indicating that studies conducted in China report significantly lower insulin outcomes compared to the USA.

Study design significantly explained the heterogeneity in waist circumference values. The adjusted R^2^ of 77.4% indicated that a large portion of the variation was due to differences in study design (e.g., randomized vs. non-randomized). Specific comparisons (non-randomized vs. randomized) showed significant differences (−2.55, 95% CI: −4.33, −0.77).

RoB is a significant predictor of heterogeneity in waist circumference and weight. The adjusted R^2^ values indicate that RoB explains a substantial portion of the heterogeneity in waist circumference (62%) and to a lesser extent weight (12.5%). Serious vs. low RoB shows a significant negative effect on weight (−4.99, 95% CI: −9.80, −0.18) and waist circumference (−2.51, 95% CI: −4.69, −0.33), indicating that studies with a higher RoB (serious) report significantly lower outcomes than those with a low RoB.

Fasting hours significantly explain the heterogeneity in total cholesterol and LDL concentrations, with adjusted R^2^ values of 31% and 45.4%, respectively. This suggests that a moderate portion of the variability in these outcomes is due to differences in fasting protocols across studies.

A one-unit change in fasting hours shows a positive effect on cholesterol (0.27, 95% CI: 0.05, 0.50) and LDL (0.34, 95% CI: 0.09, 0.59) concentrations, suggesting that longer fasting periods are associated with higher cholesterol and LDL concentrations.

While the *p*-values are not statistically significant, the adjusted R^2^ values suggest that BMI (pooled mean) explains a considerable portion of the heterogeneity in diastolic blood pressure (DBP, 48.9%) and systolic blood pressure (SBP, 28.6%). This indicates that BMI is likely an important factor influencing these outcomes. 

### 3.9. Effect of Risk of Bias on Results

The subgroup analysis by risk of bias (RoB) demonstrated that study quality might have influenced the magnitude of effects and the consistency of the results across several outcomes. While the overall findings suggest significant effects on parameters such as total cholesterol, weight, and insulin concentrations, the level of RoB appeared to affect the degree of heterogeneity observed. For example, studies categorized as having a serious RoB showed higher variability, particularly in cholesterol concentrations (*I*^2^ = 95.7%, *p* = 0.000), and in some cases, larger effect sizes were observed. However, studies with moderate and low RoB generally showed more consistent results with lower heterogeneity, as seen in waist circumference (*I*^2^ = 0.0%, *p* = 0.376) and HbA1C (*I*^2^ = 0.0%, *p* = 0.936) (Supplemental Figures S6–S19).

## 4. Discussion

The main purpose of the present study was to highlight the beneficial effects of TRE in healthy adults or in subjects with cardiometabolic disorders, associated or not with obesity. A systematic review and meta-analysis of 27 randomized and non-randomized clinical studies was performed, and the results showed that TRE is indeed beneficial, leading to weight loss and improvement in cardiovascular risk factors. The participants who followed the TRE showed a significant decrease in body weight and BMI, as well as in whole-body fat mass, lean mass and waist circumference. In addition, they showed significant improvement in cardiometabolic risk factors, such as a significant decrease in serum insulin, total cholesterol, triglycerides and LDL concentrations. However, hip circumference, total glucose, and HDL concentrations did not show significant changes. In addition, there was a slight non-significant increase in systolic and diastolic blood pressure.

As far as the heterogeneity among studies is concerned, region, study design, RoB, and fasting hours were significant predictors of heterogeneity for several key outcomes. These factors explain a large proportion of the variability between studies, as shown by the high adjusted R^2^ values for insulin, HbA1C, waist circumference, cholesterol, and LDL. These findings suggest that differences in geographic location, study methodology, risk of bias, and fasting protocols contribute substantially to the observed heterogeneity. Therefore, future studies should carefully consider and report these factors to ensure more consistent results across studies. While some variables like country and study design are highly influential for specific outcomes (insulin, HbA1C, waist circumference), other factors like age, health status and study duration do not significantly contribute to the heterogeneity for most outcomes, as evidenced by their non-significant *p*-values and low R^2^ values.

**Figure 10:**
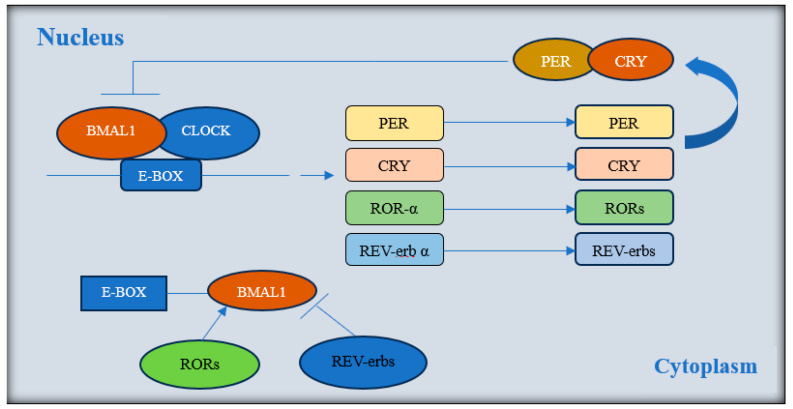
Transcription–translation feedback loops. Abbreviations: BMAL1 (brain and muscle arnt-like), CLOCK (circadian locomotor output cycles kaput), PER (period ortholog), CRY (cryptochrome), ROR (retinoic acid-related orphan receptors), REV-erb (reverse c-erb).

The benefits of TRE are based on harmonizing the eating schedule with the circadian rhythm. At the cellular level, the circadian rhythm relies on transcription–translation feedback loops that regulate the expression of key transcription factors for important clock genes. The CLOCK-BMAL1 (brain and muscle Arnt-like protein-1) transcription factor dimer leads to the expression of Period ortholog (Per1, Per2, and Per3) and Cryptochrome (Cry1 and Cry2) genes [46] (Figure 10).

Disturbances that may occur in Clock genes affect metabolic pathways related to carbohydrate and lipid metabolism resulting in hyperglycemia, insulin resistance, visceral fat accumulation, dyslipidemia, and arterial hypertension, clinical entities seen in metabolic syndrome [47,48]. In the study by Turek et al. [49], homozygous mice with loss of the Clock gene demonstrated overeating and obesity, and developed metabolic syndrome with hyperleptinemia, hyperlipidemia, hyperglycemia, insufficient insulin secretion, and steatosis of the liver. In the study by McDearmon et al. [50], Bmal1−/− knock-out mice did not follow the circadian rhythm and displayed reduced physical activity and body weight as well as reduced life expectancy. Furthermore, Bmal1−/− knock-out mice showed disruption in the expression of genes related to glucose regulation, resulting in inability of the liver to extract glucose at the required time intervals, which led to hypoglycemia during fasting hours [51].

TRE refers to a nutritional intervention based on fasting that lasts 12–16 h per day and aims to maintain the circadian rhythm at normal levels, thereby favoring metabolic homeostasis. More specifically, TRE restores normal circadian rhythms of glucose and lipid metabolism, as well as mitochondrial function, while at the same time regulating leptin and adiponectin secretion. It is extremely important that these effects are observed regardless of changes in the quantity and quality of diet and physical activity. In addition, individuals with pre-diabetes showed a reduction in appetite and an improvement in tissue sensitivity to insulin, blood pressure, and oxidative stress [52]. In a study by Hatori et al. [14], an attempt was made to compare a group of rodents that followed a free diet and a group with a restricted feeding window of 8–10 h per day. According to the results, the rodents that were fed during the active phase of 24 h with a range of 8–10 h did not show obesity, hyperinsulinemia, or steatosis of the liver, while the expression of the corresponding circadian genes, nutrient management, and daily energy expenditure improved. Recent meta-analyses in subjects with metabolic disorders have also demonstrated the effectiveness of TRE in weight loss and improvement of cardiometabolic risk factors [53,54,55].

There are few recent studies with which we share similar inclusion and exclusion criteria, like fasting duration, and we have reached similar results. The systematic review and meta-analysis by Shinje Moon et al. [17] in 2020 concluded that TRE was a promising therapeutic strategy for controlling body weight and improving metabolic dysfunction. Specifically, body weight [MD: −0.90 (95% CI: −1.71 to −0.10)] and fat mass [MD: −1.58 (95% CI: −2.64 to −0.51)] were significantly reduced, alongside systolic blood pressure [MD: −3.07 (95% CI: −5.76 to −0.37)], fasting glucose concentration [MD: −2.96 (95% CI: −5.60 to −0.33)], and triglyceride concentrations [MD: −11.60 (95% CI: −23.30 to −0.27)]. However, LDL and HDL concentrations did not show significant differences. In the systematic review and meta-analysis by Lili Liu et al. [18] in 2022, TRE significantly decreased body weight [MD: −1.60 (95% CI: −2.27 to −0.93)], fat mass [MD: −1.48 (95% CI: −1.59 to −1.38)], triglycerides [MD: −12.71 (95% CI: −24.9 to −0.52)], total cholesterol [MD: −6.45 (95% CI: −7.40 to −5.49)] and LDL concentrations [MD: −7.0 (95% CI: −9.74 to −4.24)]. However, TRE had no significant effects on waist circumference, body mass index, glycosylated hemoglobin, and blood pressure in this study. Meanwhile, our study provided additional valuable data concerning the beneficial effect of TRE on reducing BMI and waist circumference as well as improving serum insulin, total cholesterol, triglycerides and LDL concentrations. However, glucose concentrations, HDL concentrations, and blood pressure did not show significant changes in our study.

This systematic review and meta-analysis is limited to clinical studies where the participants followed a specific pattern in their eating schedule, mostly the 16:8 schedule, excluding studies with complete abstinence from food for a long period of time (for example, 24 h) or combined intermittent fasting with caloric restriction or religious fasting. A key strength of our meta-analysis is the inclusion of recent clinical trials spanning a wide range of participant characteristics, study durations, and fasting protocols. Our analysis extends prior work by including the most up-to-date evidence on the 16:8 TRE regimen, providing a comprehensive evaluation of its effects on body composition and cardiometabolic outcomes.

This study has some limitations. After thorough research, it was not possible to locate any clinical studies of the application of TRE in children or adolescents in order to review their results. In addition, there was heterogeneity in the characteristics of the populations included in the intervention groups. More specifically, the clinical study populations included men and women of different age groups, with different lifestyles and exercise habits who were either healthy or had a metabolic disorder, such as obesity, diabetes, or metabolic syndrome. Another factor that makes it difficult to draw firm conclusions concerns the fact that the duration of the intervention in weeks varied significantly among clinical studies, as well as the number of participants in the intervention and control groups. In light of the identified risks of bias, unmeasured confounders in non-randomized studies may have contributed to the observed effects, and therefore the results should be interpreted with caution. Moreover, due to the potential of publication bias, the results for outcomes like BMI and waist circumference should also be interpreted with caution due to the small number of included studies and the potential for missing smaller or negative studies that could affect the pooled estimates.

Our findings suggest that risk of bias may have influenced the magnitude of effects observed in some outcomes, particularly those with higher heterogeneity, such as cholesterol and insulin concentrations. While studies with serious RoB exhibited greater variability, overall trends were consistent across studies with different risk profiles. Stratified analyses and meta-regressions showed that the impact of RoB was statistically significant for outcomes such as waist circumference and weight, suggesting a need for cautious interpretation of these results. Nonetheless, the consistency of findings across different quality studies adds robustness to the conclusion that TRE leads to meaningful improvements in body weight, cardiometabolic risk factors, and lipid profiles. Future research should aim to minimize potential biases by adopting more rigorous methodologies.

## 5. Conclusions

In conclusion, TRE leads to a decrease in body weight and total body fat mass and improves cardiometabolic risk factors without calorie restriction. However, more clinical studies should be carried out with a variety of participants, including children and adolescents, and a longer duration of intervention in order to draw safer conclusions.

## Figures and Tables

**Table 1:** Summary of the 27 studies included in the systematic review and meta-analysis.

1st Author‘s Name	Publication Year	Study Design	Study Duration	TRE Regimen	Total Participants	Sex	Participants Characteristics	Number of Participants Per Group
Dylan A. Lowe [19]	2020	RCT	12 weeks	16:8	116	Female = 46 Male = 70	men and women, 18 to 64 years with a BMI of 27 to 43 kg/m^2^	Intervention group: 59 Control group: 57
Nicholas Edward Phillips [20]	2021	RCT	6 months	12:12	45	Female = 32 Male = 13	adults with a body mass index BMI ≥ 20 kg/m^2^, stable weight (±2 kg) over the previous 3 months and at least one component of MS	Intervention group: 25 Control group: 20
Tingting Che [21]	2021	RCT	12 weeks	10:14	120	Female = 55 Male = 65	overweight adults with type 2 diabetes	Intervention group: 60 Control group: 60
Sek Ying Chair [22]	2022	RCT	3 weeks	16:8	67	Female = 36 Male = 31	overweight and obese adults with prediabetes	Intervention group: 33 Control group: 34
Tatiana Moro [23]	2016	RCT	8 weeks	16:8	17	Male = 17	male middle- and long-distance runners	Intervention group: 10 Control group: 7
Christopher J. Kotarsky [24]	2021	RCT	8 weeks	16:8	21	Female = 18 Male = 3	Physically inactive and overweight or obese female and male participants, determined by a BMI between 25.0 and 34.9 kg/m^2^, between the ages of 35 and 60 years	Intervention group: 11 Control group: 10
Hua Cai [25]	2019	RCT	4 weeks	16:8	174	Female = 145 Male = 52	adults with NAFLD	Intervention group: 95 Control group: 79
Eduard Isenmann [26]	2021	RCT	14 weeks	16:8	35	Female = 21 Male = 14	healthy, physically active, between 20 and 40 years old, BMI of less than 33 kg/m^2^	Intervention group: 18 Control group: 17
Przemysław Domaszewski [27]	2020	RCT	6 weeks	16:8	45	Female = 45	non-smoking women over 60 years of age	Intervention group: 25 Control group: 20
Grant M Tinsley [28]	2019	RCT	8 weeks	16:8	40	Female = 40	resistance-trained healthy females 18–30 years	Intervention group: 13 Control group: 14
Aidan J. Brady [29]	2020	RCT	8 weeks	16:8	23	Male = 23	male middle- and long-distance runners	Intervention group: 12 Control group: 11
Grant M. Tinsley [30]	2017	RCT	8 weeks	20:4	18	Male = 18	healthy, active men who had not followed a consistent RT program over the previous three months	Intervention group: 10 Control group: 8
Robert Jones [31]	2020	non-RCT	2 weeks	16:8	16	Male = 16	healthy males	Intervention group: 8 Control group: 8
Tatiana Moro [32]	2020	RCT	4 weeks	16:8	16	Male = 16	healthy young men from 5 different elite cyclist teams	Intervention group: 8 Control group: 8
Shuhao Lin [33]	2023	RCT	12 months	16:8	60	Female = 50 Male = 10	adults with obesity	Intervention group: 30 Control group: 30
Emily N.C. Manoogian [34]	2022	RCT	12 weeks	10:14	137	Female = 12 Male = 125	healthy adults	Intervention group: 70 Control group: 67
Vasiliki Pavlou [35]	2023	RCT	6 months	16:8	75	Female = 53 Male = 22	adults with type 2 diabetes	Intervention group: 25 Control group: 25
Jéssica D. Schroder [36]	2021	non-RCT	3 months	16:8	32	Female = 32	obese women (BMI ≥ 30 kg/m^2^)	Intervention group: 20 Control group: 12
Naseer Ahmed [37]	2021	non-RCT	6 weeks	12:12	35	Female = 15 Male = 20	age of 20–70 years, with serum HDL< 40 mg/dL for men and <50 mg/dL for women	Intervention group: 15 Control group: 20
Ashley P. Tovar [38]	2021	randomized crossover	4 weeks	16:8	15	Male = 15	healthy, endurance trained male runners between 21–36 years of age	Intervention group: 8 Control group: 7
Elizabeth F. Sutton [39]	2018	randomized crossover	5 weeks	18:6	8	Male = 8	male with prediabetes	8 individuals
Evelyn B. Parr [40]	2020	randomized crossover	5 days	16:8	11	Male = 11	men (aged 30–45 years) with overweight/obesity and inactive/sedentary lifestyle	11 individuals
Humaira Jamshed [41]	2019	randomized crossover	4 days	18:6	11	Female = 4 Male = 7	adults aged 20–45 years old with BMI between 25.0 kg and 35.0 kg/m^2^, a body weight between 68 kg and 100 kg	11 individuals
Emma C. E. Meessen [42]	2022	randomized crossover	11 days	22:2	11	Female = 6 Male = 5	free-living healthy lean individuals	11 individuals
Kim S Stote [43]	2007	randomized crossover	8 weeks	20:4	15	Female = 10 Male = 5	healthy men and women aged 40–50 years	15 individuals
Christine E. Richardson [44]	2023	randomized crossover	4 weeks	16:8	15	Male = 15	endurance-trained male runners	15 individuals
Michael J. Wilkinson [45]	2020	single-arm, paired-sample trial	12 weeks	14:10	19	Female = 6 Male = 13	participants with metabolic syndrome	19 individuals

Abbreviations: TRE (time-restricted eating), RCT (randomized clinical trial), non-RCT (non-randomized clinical trial).

**Table 2:** Meta-analyses with clinical trials compared to baseline.

Outcome	No. of Studies [Reference]	Type of Model	MD (95% CI)	*p*-Value	Heterogeneity *I*^2^%	*p*-Value
Body weight	[23]	Random	−1.622 kg (−2.302 to −0.941)	*p* < 0.0001	96.1%	*p* < 0.0001
BMI	[11]	Random	−0.919 kg/m^2^ (−1.189 to −0.650)	*p* < 0.0001	82%	*p* < 0.0001
WBFM	[17]	Fixed	−0.662 kg (−0.795 to −0.530)	*p* < 0.0001	17.8%	*p* = 0.246
LM	[9]	Fixed	−0.448 kg (−0.672 to −0.224)	*p* < 0.0001	0.0%	*p* = 0.983
Waist circumference	[10]	Random	−2.015 cm (−3.212 to −0.819)	*p* = 0.001	68.4%	*p* = 0.001
Hip circumference	[3]	Fixed	−0.440 cm (−1.432 to 0.552	*p* = 0.385	31.6%	*p* = 0.232
Waist–hip ratio	[1]	Fixed	0.006 cm (−0.020 to 0.032)	*p* = 0.651		
Total body water	[2]	Fixed	0.372 kg (−0.246 to 0.990)	*p* = 0.238	3.8%	*p* = 0.308
SBP	[10]	Random	−0.212 mmHg (−2.721 to 2.298)	*p* = 0.869	71.8%	*p* < 0.0001
DBP	[10]	Random	0.466 mmHg (−1.207 to 2.140)	*p* = 0.585	62%	*p* = 0.005
Insulin	[13]	Random	−0.458 mIU/L (−0.843 to −0.073)	*p* = 0.020	92.1%	*p* < 0.0001
HbA1C	[7]	Random	−0.175% (−0.569 to 0.219)	*p* = 0.385	98.7%	*p* < 0.0001
Glucose	[17]	Fixed	0.124 mg/dL (−0.193 to 0.442)	*p* = 0.444	24.9%	*p* = 0.167
Total cholesterol	[15]	Random	−2.889 mg/dL (−5.447 to −0.330)	*p* = 0.027	95.5%	*p* < 0.0001
HDL	16	Random	0.632 mg/dL (−0.636 to 1.899)	*p* = 0.329	98.7%	*p* < 0.0001
LDL	[14]	Random	−2.717 mg/dL (−4.412, −1.021)	*p* = 0.002	94.7%	*p* < 0.0001
Triglycerides	[16]	Random	−3.782 mg/dL (−6.180 to 1.384)	*p* = 0.002	88.2%	*p* < 0.0001

Abbreviations: MD (median), 95% CI (95% confidence interval), BMI (body mass index), WBFM (whole-body fat mass), LM (lean mass), SBP (systolic blood pressure), DBP (diastolic blood pressure), HbA1C (hemoglobin A1C), HDL (high-density lipoprotein), LDL (low-density lipoprotein).

**Table 3:** Heterogeneity assessments via meta-regression models.

Factors	ΒΜΙ	Weight	Waist Circumference	Insulin	HbA1C	SBP	DBP	Cholesterol	HDL	LDL	Triglycerides
Country (USA, European countries, Australia, China, Brazil)	*p* = 0.855	*p* = 0.744	*p* = 0.825	*p* = 0.005; adj R^2^ = 92.9%	*p* = 0.006; adj R^2^ = 94.2%	*p* = 0.811	*p* = 0.929	*p* = 0.595	*p* = 0.756	*p* = 0.813	*p* = 0.372
Study duration, in weeks	*p* = 0.464	*p* = 0.765	*p* = 0.659	*p* = 0.911	*p* = 0.774	*p* = 0.960	*p* = 0.213; adj R^2^ = 16.0%	*p* = 0.917	*p* = 0.755	*p* = 0.892	*p* = 0.338
Female %	*p* = 0.705	*p* = 0.501	*p* = 0.591	*p* = 0.916	*p* = 0.712	*p* = 0.609	*p* = 0.342	*p* = 0.562	*p* = 0.891	*p* = 0.316	*p* = 0.380
Age, in years (pooled mean)	*p* = 0.565	*p* = 0.516	*p* = 0.646	*p* = 0.494	*p* = 0.459	*p* = 0.483	*p* = 0.730	*p* = 0.607	*p* = 0.945	*p* = 0.922	*p* = 0.467
BMI (polled mean)	*p* = 0.508	*p* = 0.625	*p* = 0.941	*p* = 0.110; adj R^2^ = 25.2%	*p* = 0.605	*p* = 0.165; adj R^2^ = 28.6%	*p* = 0.064; adj R^2^ = 48.9%	*p* = 0.586	*p* = 0.645	*p* = 0.710	*p* = 0.191; adj R^2^ = 8.9%
Study design (randomized study, non-randomized study, crossover design)	*p* = 0.066; adj R^2^ = 25.7%	*p* = 0.208	*p* = 0.011; adj R^2^ = 77.4%	*p* = 0.580	-	*p* = 0.197; adj R^2^ = 36.7%	*p* = 0.297	*p* = 0.524	*p* = 0.286	*p* = 0.318	*p* = 0.293
Fasting hours	*p* = 0.145; adj R^2^ = 12.9%	*p* = 0.294	*p* = 0.282	*p* = 0.079; adj R^2^ = 34.1%	*p* = 0.640	*p* = 0.183; adj R^2^ = 14.0%	*p* = 0.190; adj R^2^ = 17.9%	*p* = 0.022; adj R^2^ = 31.0%	*p* = 0.996	*p* = 0.012; adj R^2^ = 45.4%	*p* = 0.670
RoB (low, moderate, serious)	*p* = 0.148; adj R^2^ = 22.5%	*p* = 0.032; adj R^2^ = 12.5%	*p* = 0.045; adj R^2^ = 62.0%	*p* = 0.581	*p* = 0.059; adj R^2^ = 46.8%	*p* = 0.907	*p* = 0.758	*p* = 0.552	*p* = 0.287	*p* = 0.465	*p* = 0.244
Population (healthy, metabolic syndrome)	*p* = 0.933	*p* = 0.586	*p* = 0.989	*p* = 0.389	*p* = 0.395	*p* = 0.355	*p* = 0.562	*p* = 0.890	*p* = 0.985	*p* = 0.785	*p* = 0.591

Note: Meta-regressions were performed for the variables with statistically significant heterogeneity. The *p*-values of the joint test for all covariates are reported. The adjusted R^2^ (proportion of between-study variance explained) is reported only for *p* < 0.20. Bold text indicates the statistically significant results. Weight: ROB, moderate vs. low: 1.01 (95% CI: −1.63, 3.65), serious vs. low: −4.99 (95% CI: −9.80, −0.18). Waist circumference: design, non-randomized vs. randomized: −2.55 (95% CI: −4.33, −0.77), ROB, moderate vs. low: 0.16 (95% CI: −1.44, 1.76), serious vs. low: −2.51 (95% CI: −4.69, −0.33). Insulin, region, Europe vs. USA: 0.82 (95% CI: −2.07, 0.43), China vs. USA: −3.27 (95% CI: −4.76, −1.78), Brazil vs. USA: −1.45 (95% CI: −3.23, −0.33); HbA1C: Region, Europe vs. USA: 0.32 (95% CI: −1.61, 2.24), China vs. USA: −4.92 (95% CI: −6.96, −2.87). Cholesterol, Fasting, 1−unit change: 0.27 (95% CI: 0.05, 0.50). Triglycerides, Fasting, 1-unit change: 0.34 (95% CI: 0.09, 0.59. Abbreviations: BMI (body mass index), HbA1C (hemoglobin A1C), SBP (systolic blood pressure), DBP (diastolic blood pressure), HDL (high-density lipoprotein), LDL (low-density lipoprotein).

## Data Availability

Not applicable.

## Supplementary Materials

The following supporting information can be downloaded at: https://www.mdpi.com/article/10.3390/nu16213700/s1, **Supplemental Materials—Funnel Plots**; Supplemental Figure S1a. Funnel plot for body weight, Supplemental Figure S1b. Funnel plot for BMI (body mass index), Supplemental Figure S1c. Funnel plot for whole-body fat mass, Supplemental Figure S1d. Funnel plot for lean mass, Supplemental Figure S1e. Funnel plot for total body water. Supplemental Figure S2a. Funnel plot for waist circumference, Supplemental Figure S2b. Funnel plot for hip circumference, Supplemental Figure S2c. Funnel plot for waist-to-hip ratio. Supplemental Figure S3a. Funnel plot for SBP (systolic blood pressure), Supplemental Figure S3b. Funnel plot for DBP (diastolic blood pressure). Supplemental Figure S4a. Funnel plot for insulin concentrations, Supplemental Figure S4b. Funnel plot for HbA1C (Hemoglobin A1C), Supplemental Figure S4c. Funnel plot for glucose concentrations. Supplemental Figure S5a. Funnel plot for total cholesterol concentrations, Supplemental Figure S5b. Funnel plot for LDL (low-density lipoprotein) concentrations, Supplemental Figure S5c. Funnel plot for triglyceride concentrations, Supplemental Figure S5d. Funnel plot for HDL (high-density lipoprotein) concentrations. **Supplemental Materials—Figures for subgroup analysis;** Figure S6. Subgroup analysis for body weight. Figure S7. Subgroup analysis for BMI (body mass index). Figure S8. Subgroup analysis for whole body fat mass. Figure S9. Subgroup analysis for lean mass. Figure S10. Subgroup analysis for waist circumference. Figure S11. Subgroup analysis for SBP (systolic blood pressure). Figure S12. Subgroup analysis for DBP (diastolic blood pressure). Figure S13. Subgroup analysis for insulin. Figure S14. Subgroup analysis for HbA1C (Hemoglobin A1C). Figure S15. Subgroup analysis for glucose. Figure S16. Subgroup analysis for cholesterol. Figure S17. Subgroup analysis for LDL (low-density lipoprotein). Figure S18. Subgroup analysis for triglycerides. Figure S19. Subgroup analysis for HDL (high-density lipoprotein)**.**
