# Supplementary material for: The Effect of Time-Restricted Eating on Cardiometabolic Risk Factors: A Systematic Review and Meta-Analysis
Source: Nutrients. 2024 Oct 30;16(21):3700. doi: 10.3390/nu16213700 (PMC11547938; doi:10.3390/nu16213700)
Supplement: Supplementary file 1 [file nutrients-16-03700-s001.zip › nutrients-3237280-Supplemental Figures-Funnel plots/nutrients-3237280-Supplemental Figures-Funnel plots-29-10-2024.pdf]

SUPPLEMENTAL MATERIAL – FUNNEL PLOTS

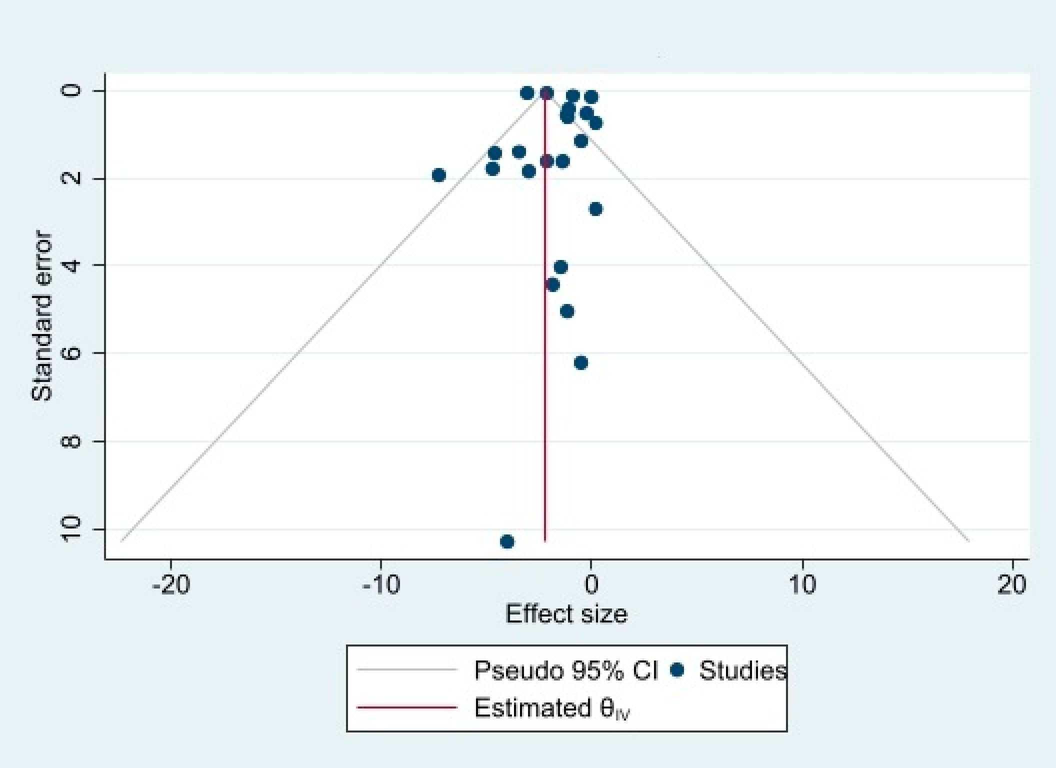

Supplemental Figure S1a: Funnel plot for body weight

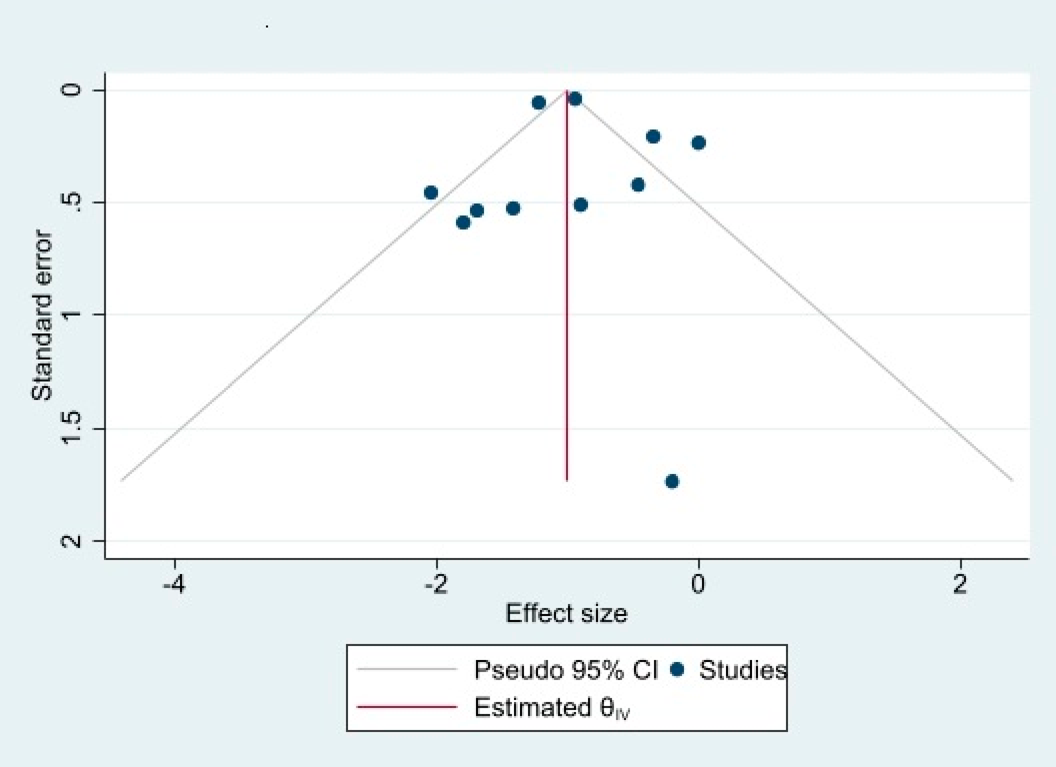

Supplemental Figure S1b: Funnel plot for BMI

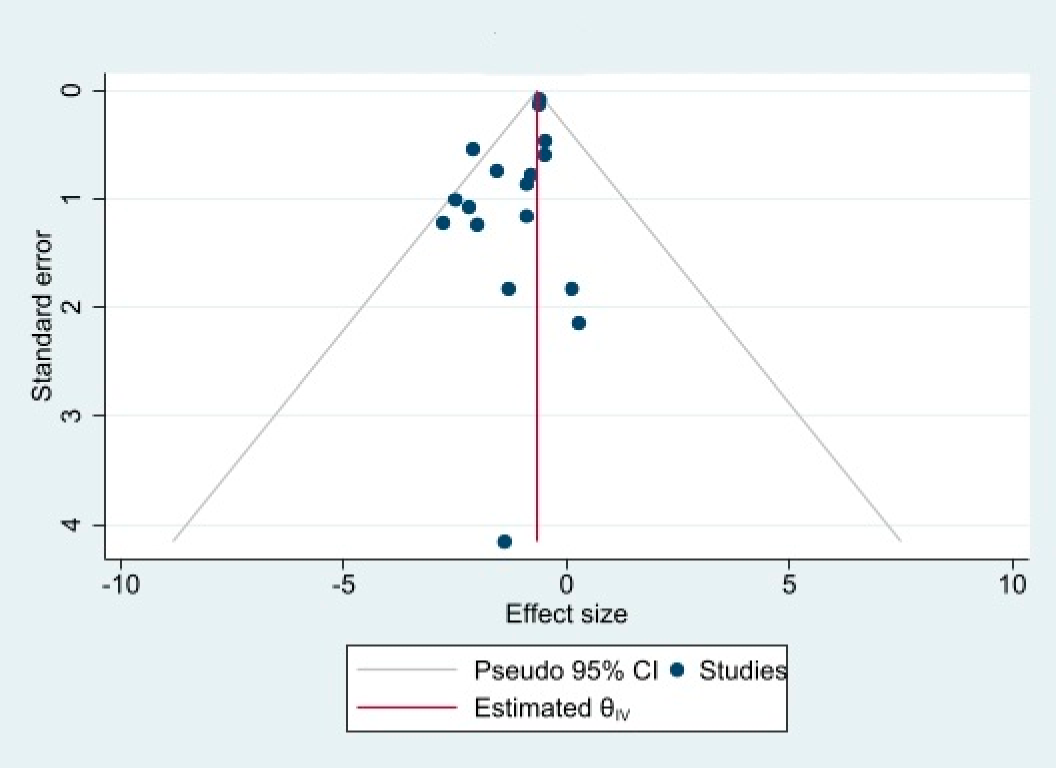

Supplemental Figure S1c: Funnel plot for whole body fat mass

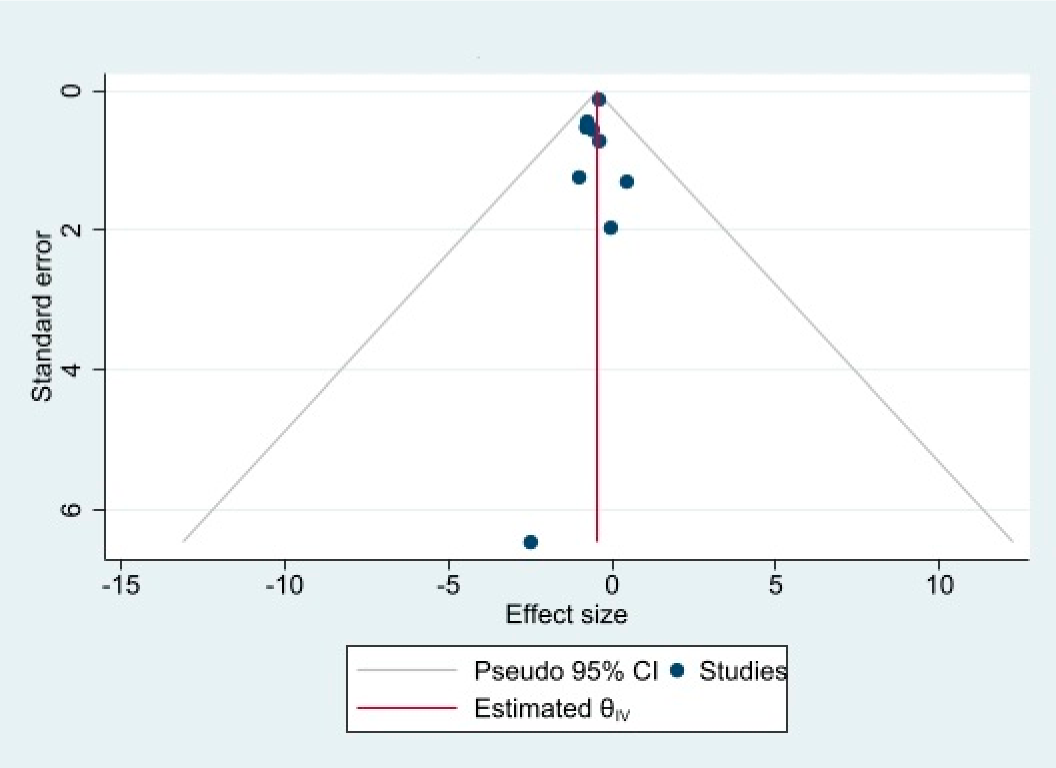

Supplemental Figure S1d: Funnel plot for lean mass

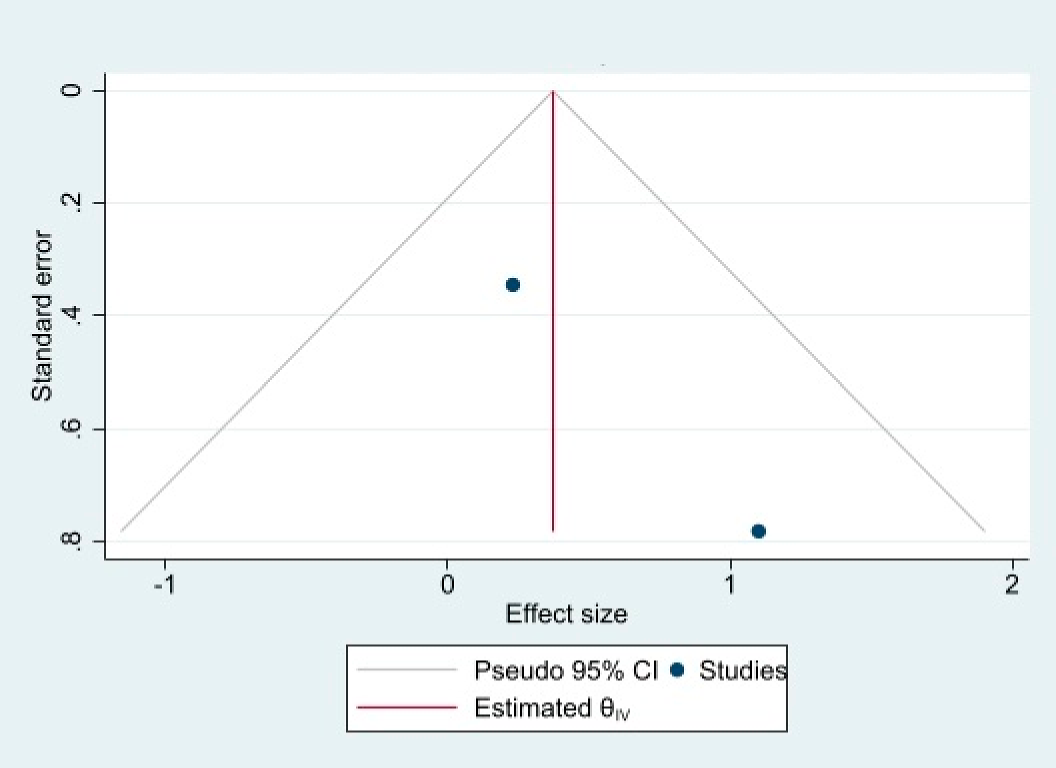

Supplemental Figure S1e: Funnel plot for total body water

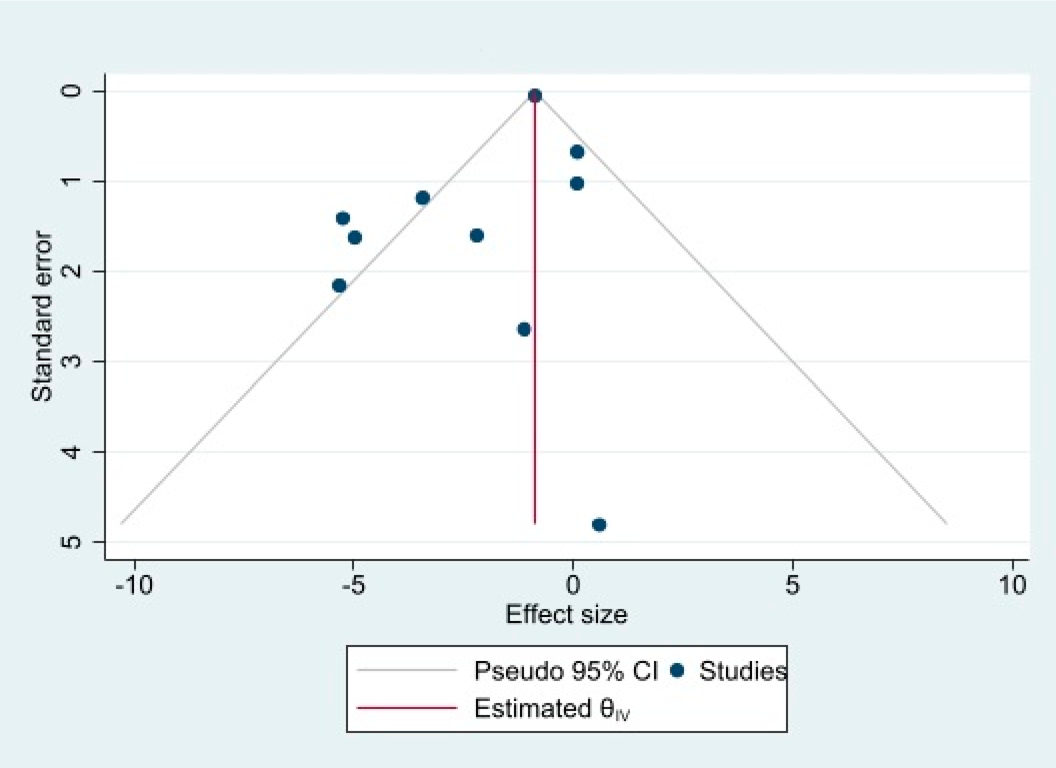

Supplemental Figure S2a: Funnel plot for waist circumference

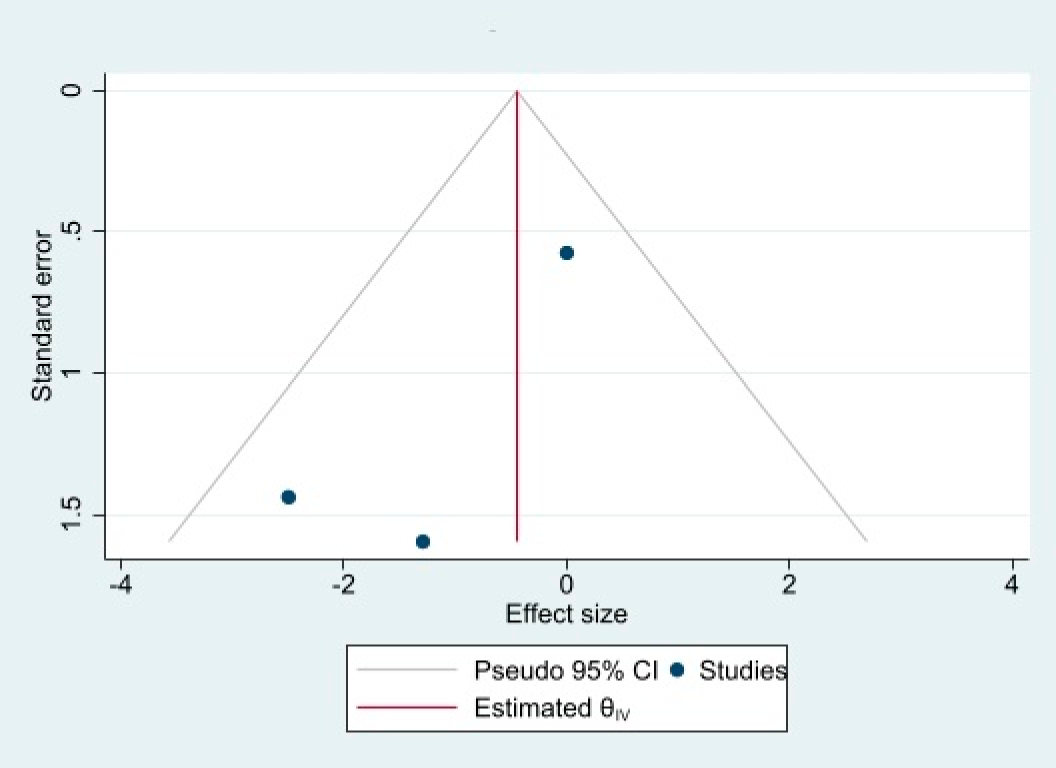

Supplemental Figure S2b: Funnel plot for hip circumference

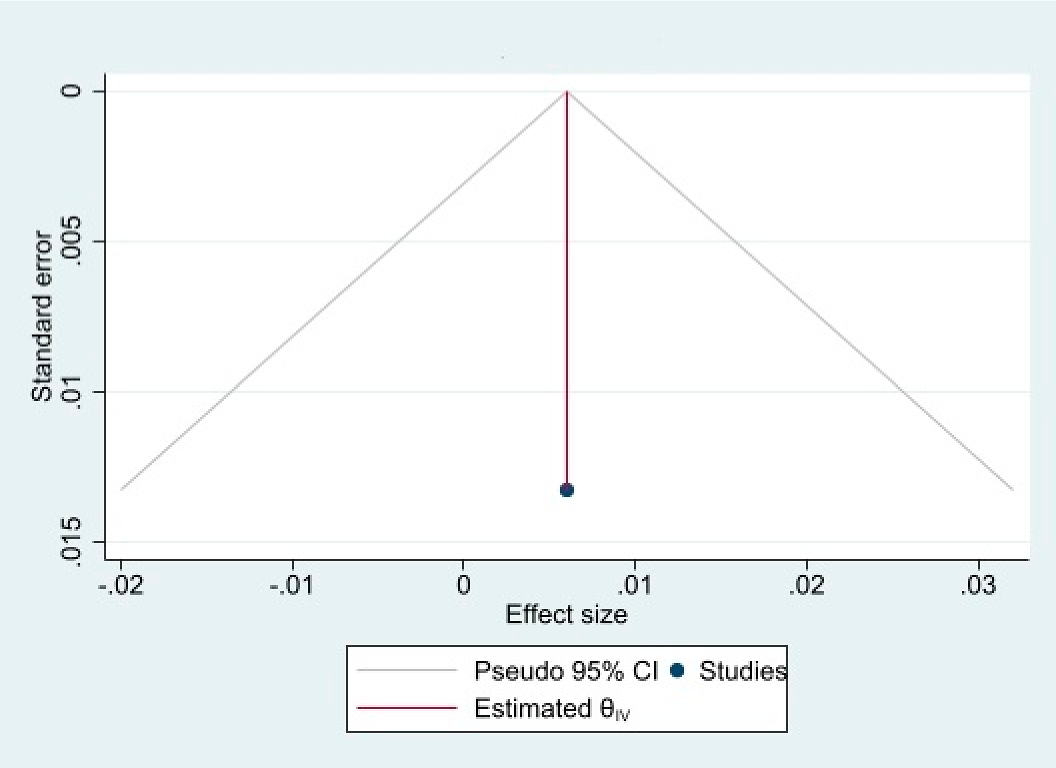

Supplemental Figure S2c: Funnel plot for waist-to-hip ratio

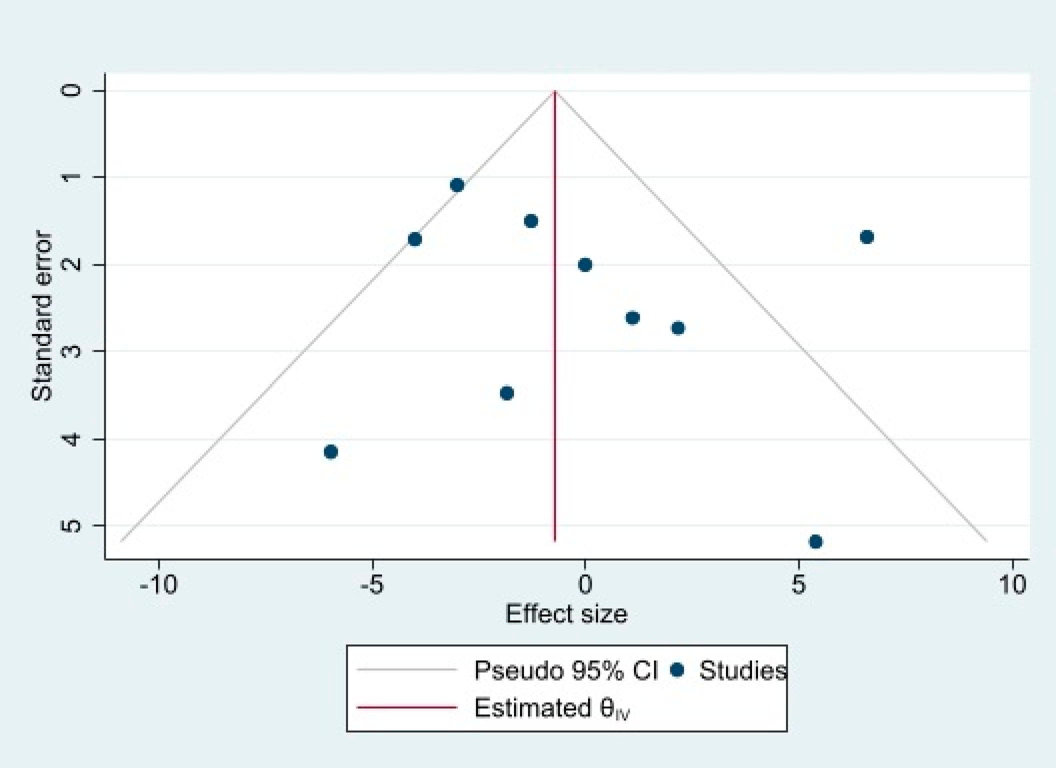

Supplemental Figure S3a: Funnel plot for systolic blood pressure

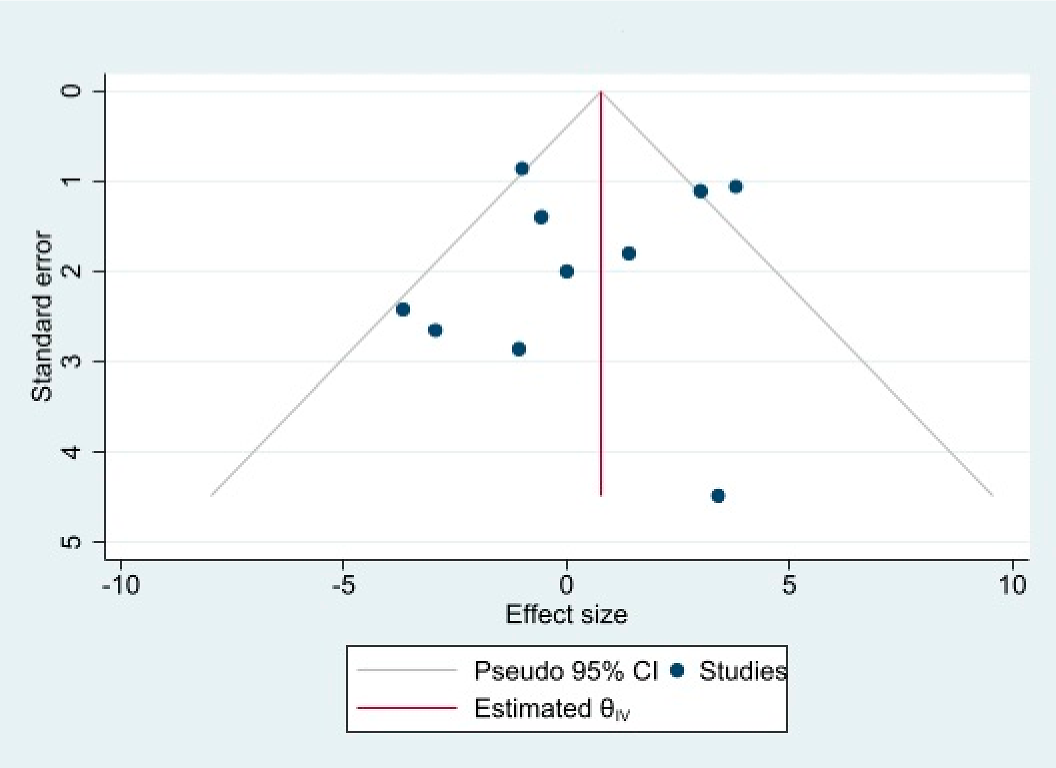

Supplemental Figure S3b: Funnel plot for diastolic blood pressure

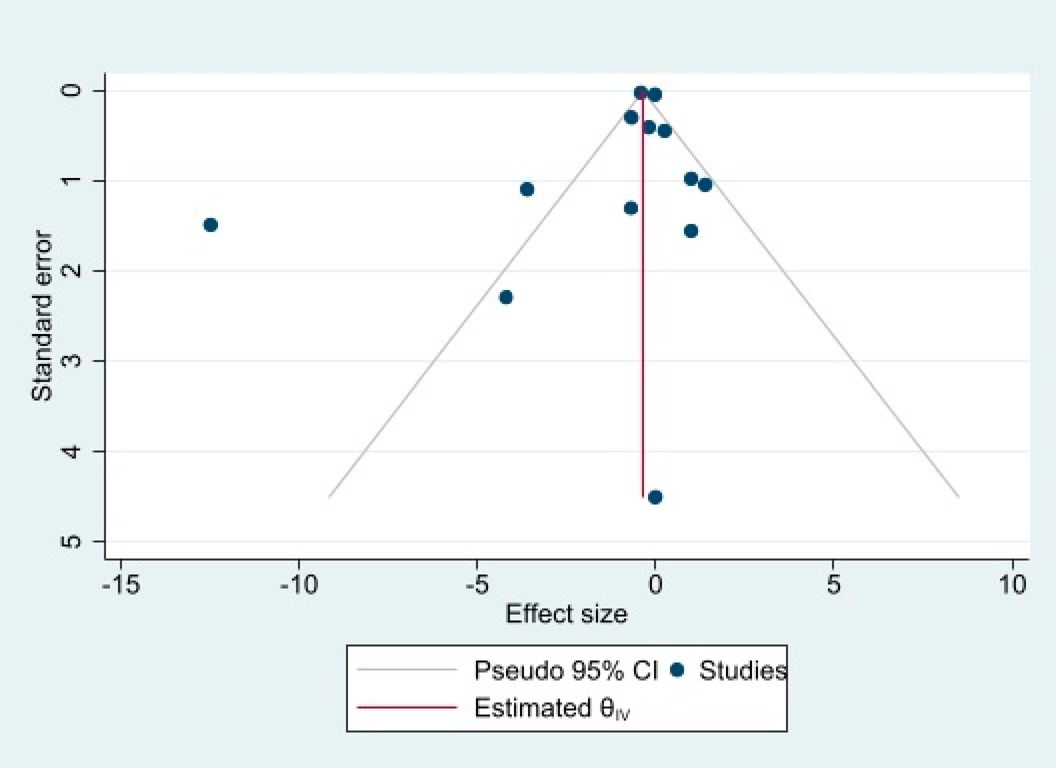

Supplemental Figure S4a: Funnel plot for insulin concentrations

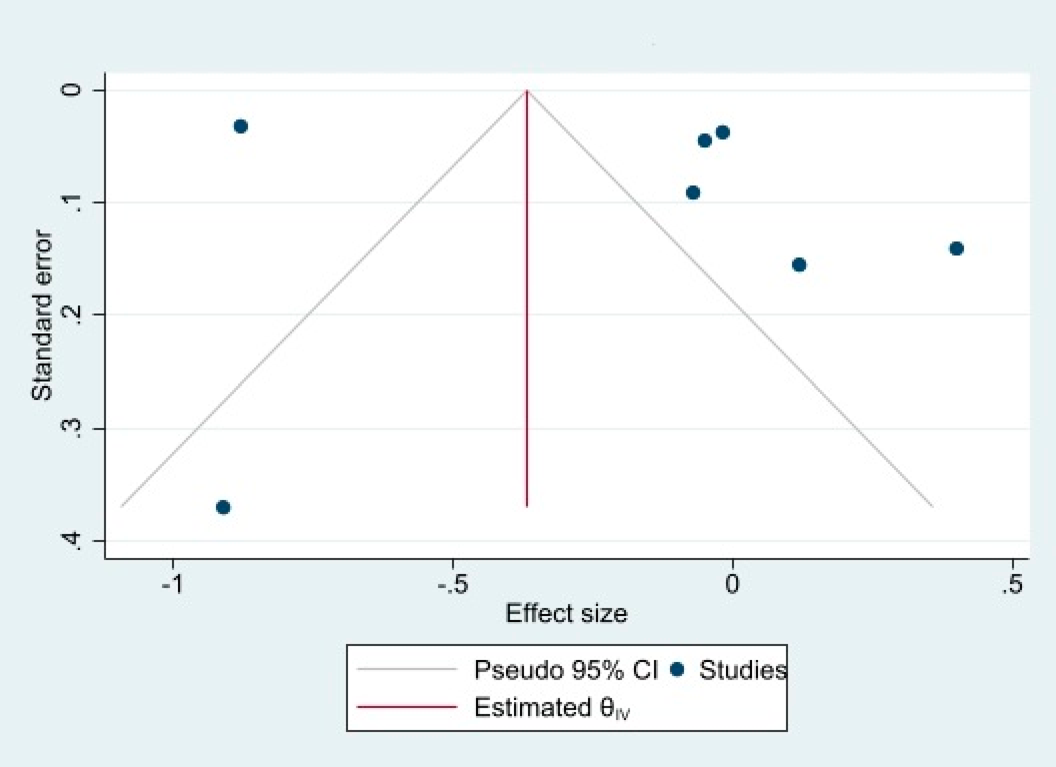

Supplemental Figure S4b: Funnel plot for HbA1C

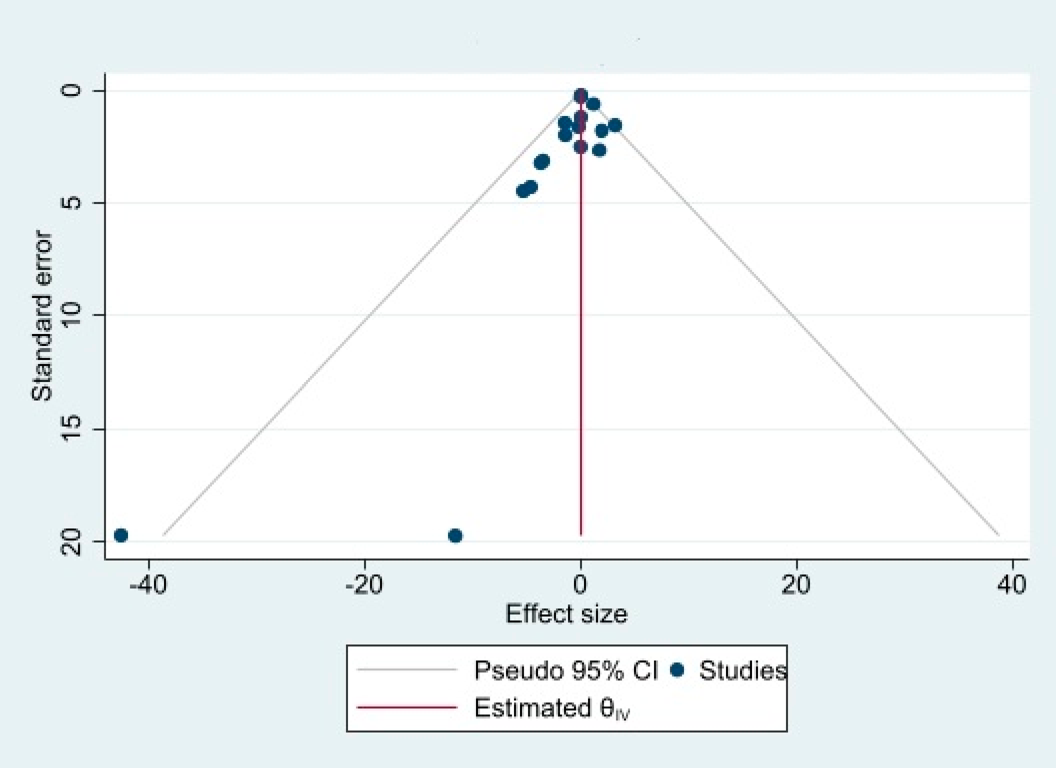

Supplemental Figure S4c: Funnel plot for glucose concentrations

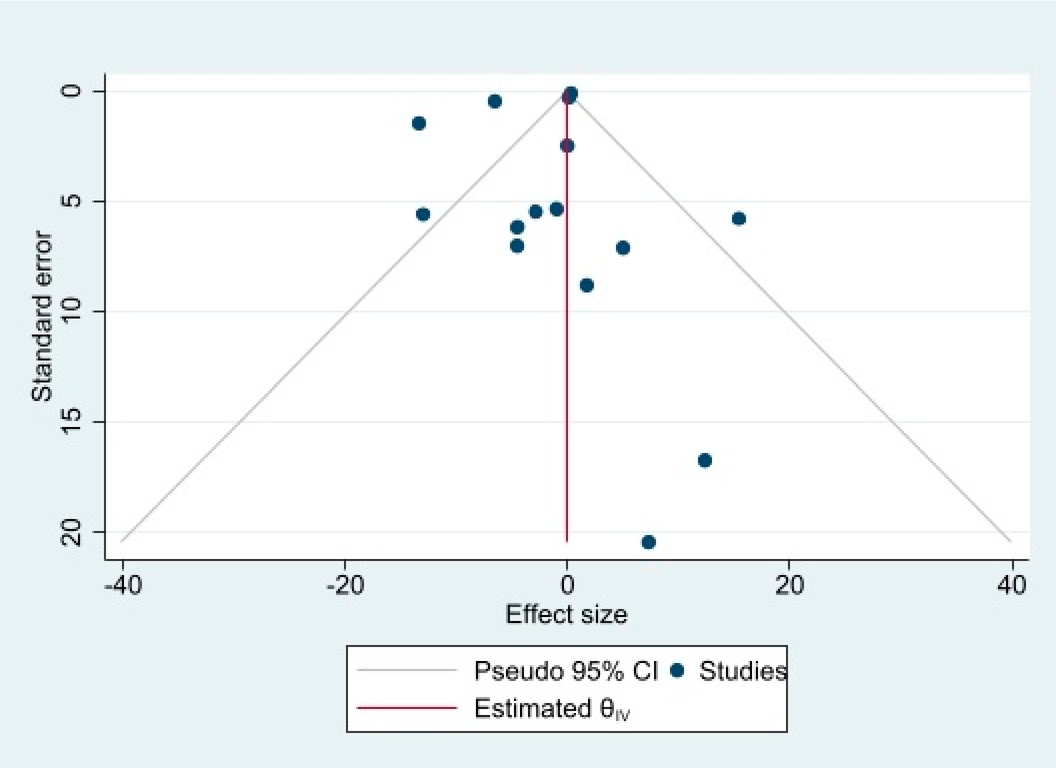

Supplemental Figure S5a: Funnel plot for total cholesterol concentrations

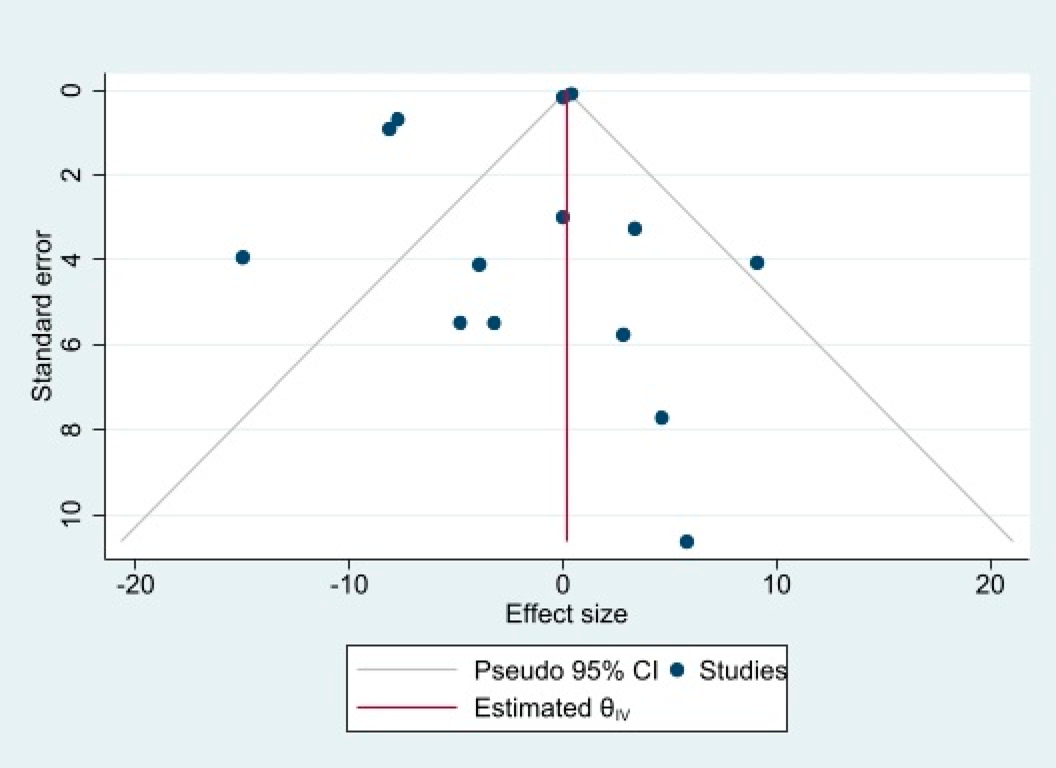

Supplemental Figure S5b: Funnel plot for LDL concentrations

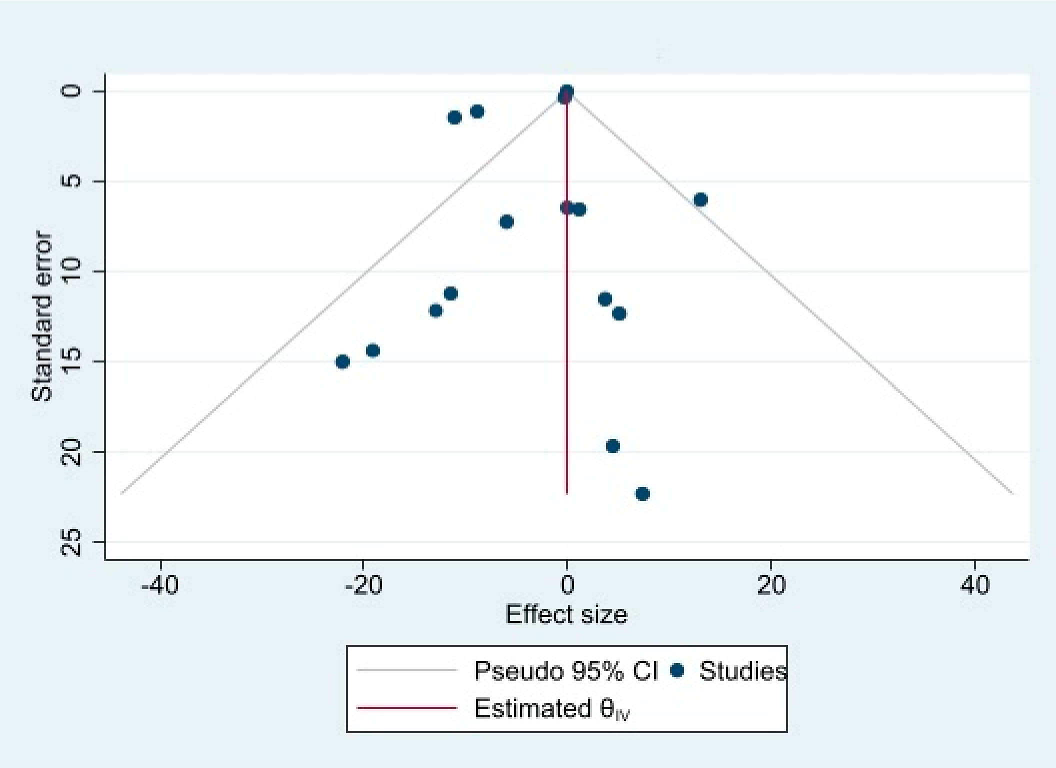

Supplemental Figure S5c: Funnel plot for triglyceride concentrations

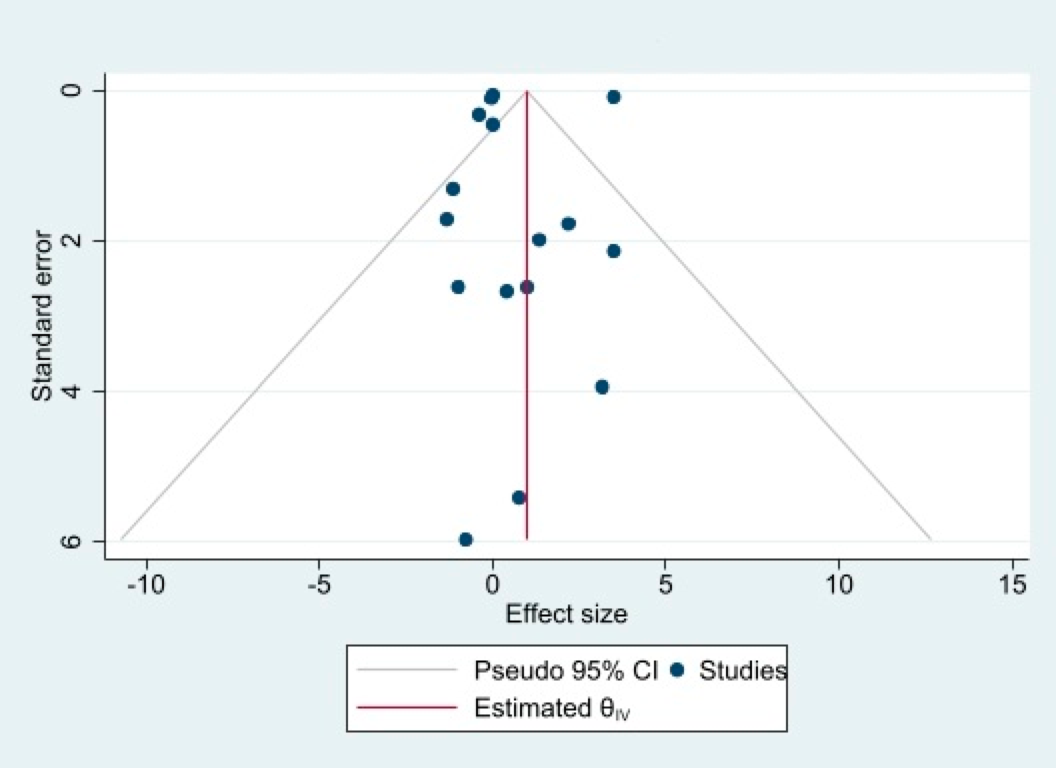

Supplemental Figure S5d: Funnel plot for HDL concentrations
