# Supplementary material for: The Effect of Time-Restricted Eating on Cardiometabolic Risk Factors: A Systematic Review and Meta-Analysis
Source: Nutrients. 2024 Oct 30;16(21):3700. doi: 10.3390/nu16213700 (PMC11547938; doi:10.3390/nu16213700)
Supplement: Supplementary file 1 [file nutrients-16-03700-s001.zip › nutrients-3237280-Supplemental Figures-Subgroup Analysis/nutrients-3237280-Supplemental Figures for Subgroup Analysis_29-10-2024.pdf]

SUPPLEMENTAL MATERIAL – FIGURES FOR SUBGROUP ANALYSIS

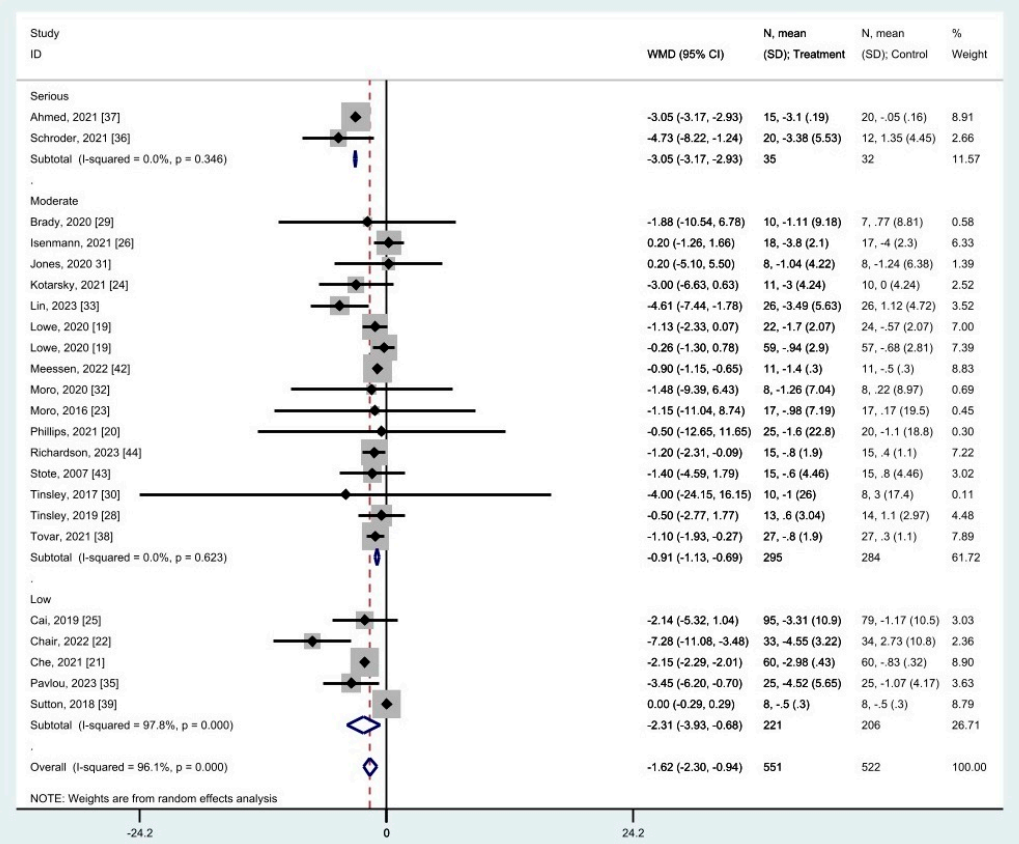

Supplemental Figure S6. Subgroup analysis for body weight by risk of bias

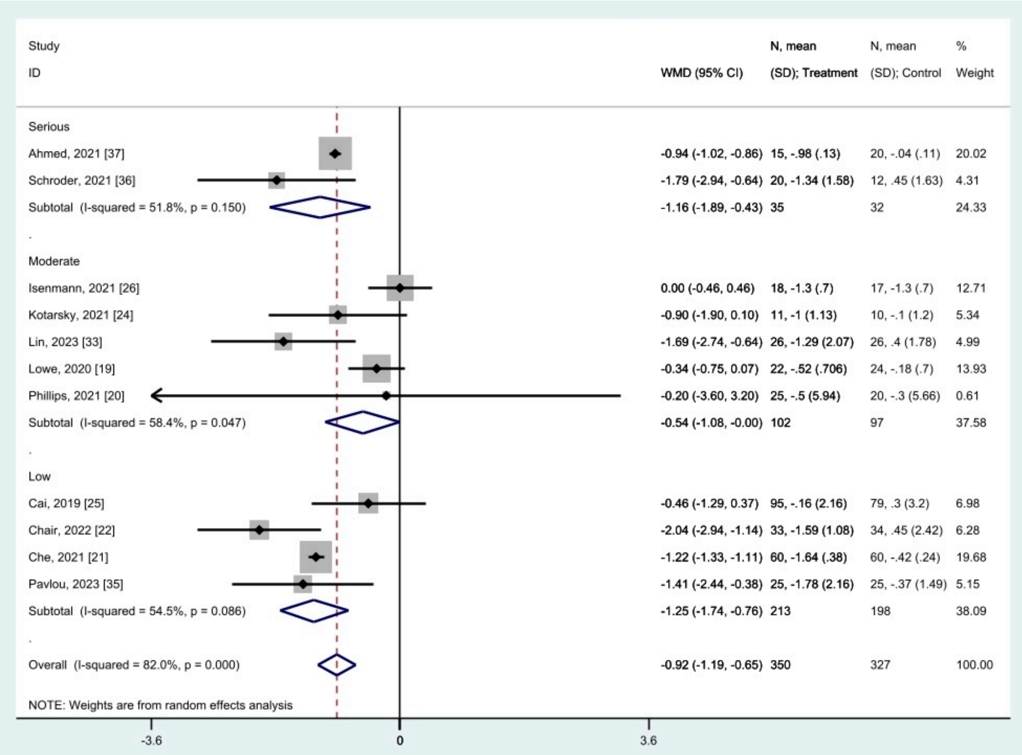

Supplemental Figure S7. Subgroup analysis for BMI by risk of bias

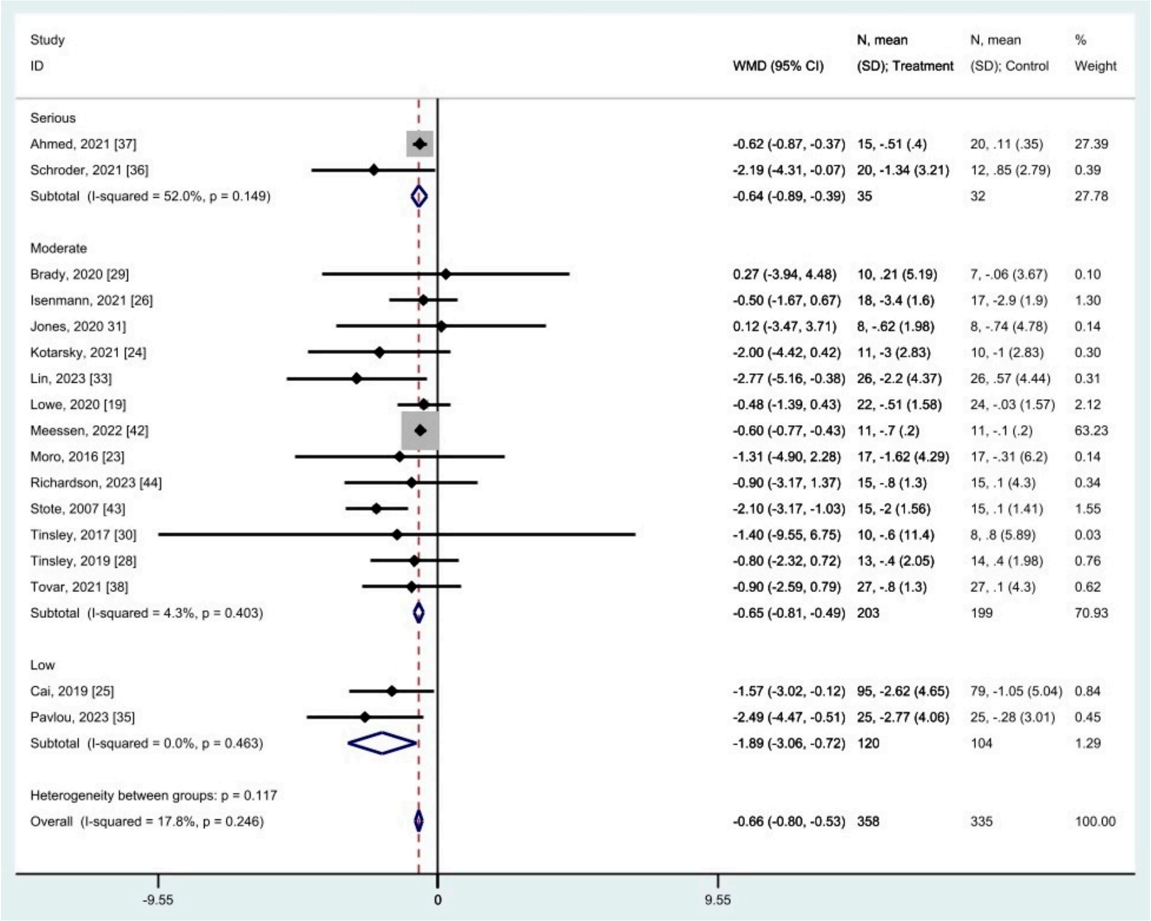

Supplemental Figure S8. Subgroup analysis for whole body fat mass by risk of bias

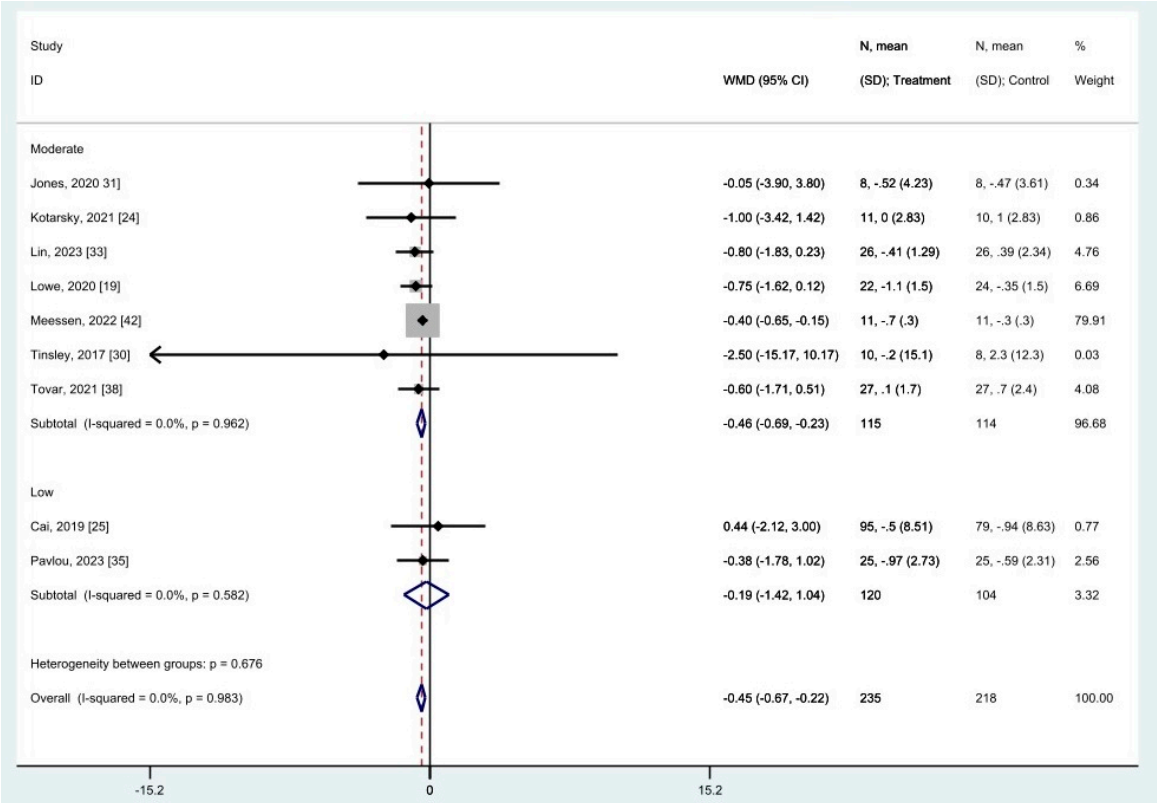

Supplemental Figure S9. Subgroup analysis for lean mass by risk of bias

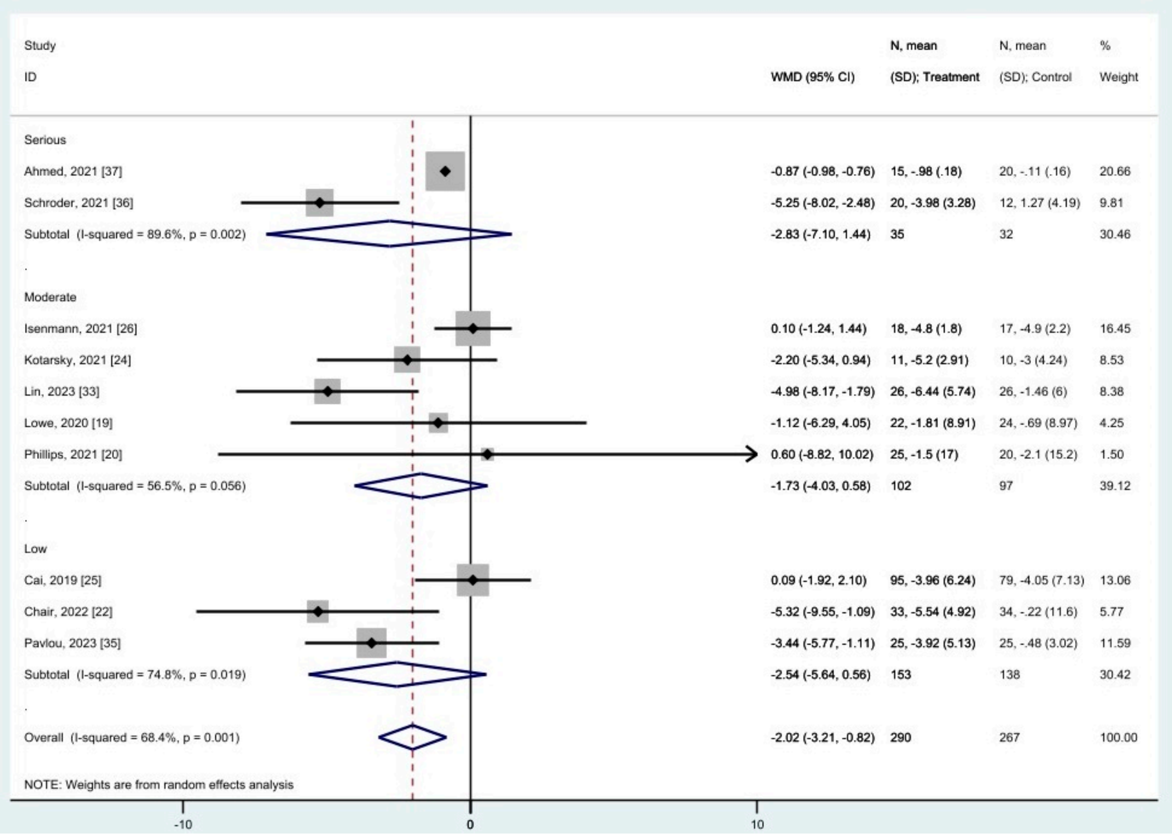

Supplemental Figure S10. Subgroup analysis for waist circumference by risk of bias

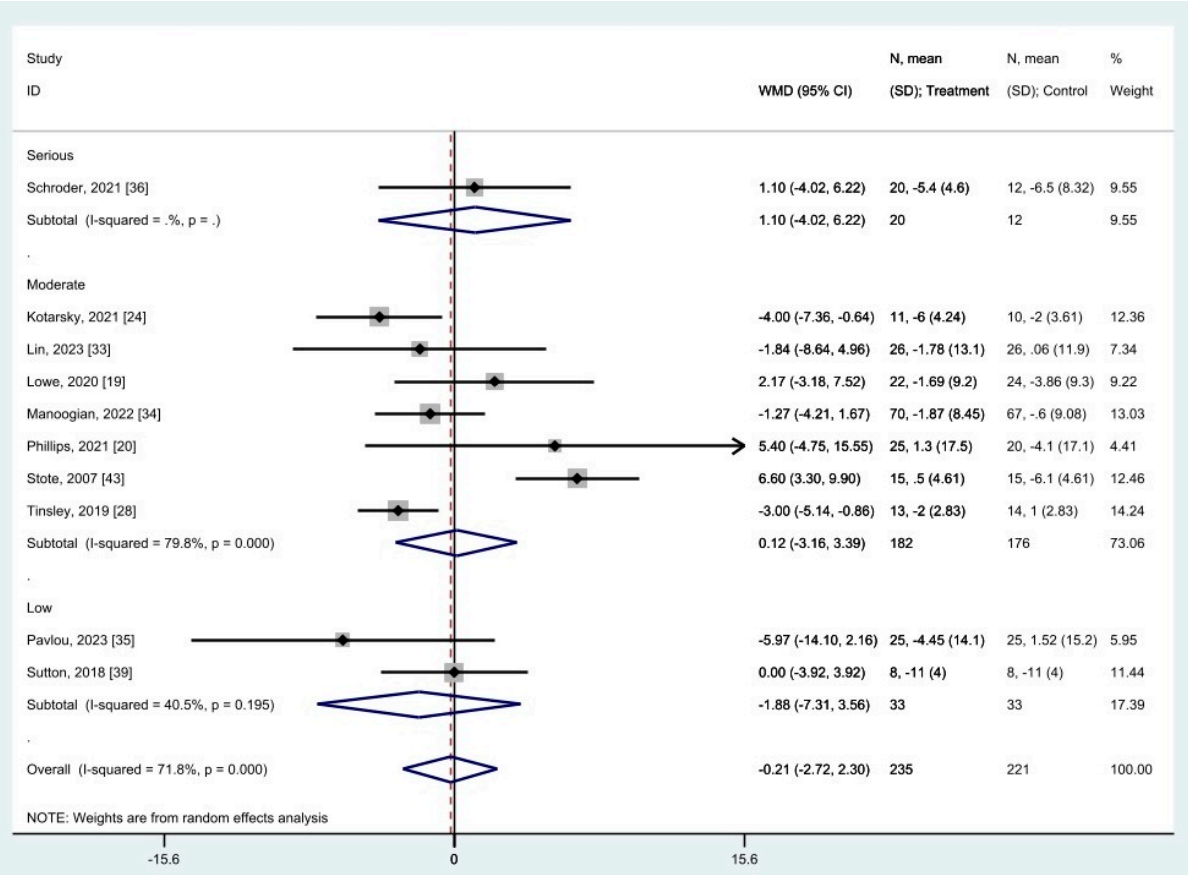

Supplemental Figure S11. Subgroup analysis for systolic blood pressure by risk of bias

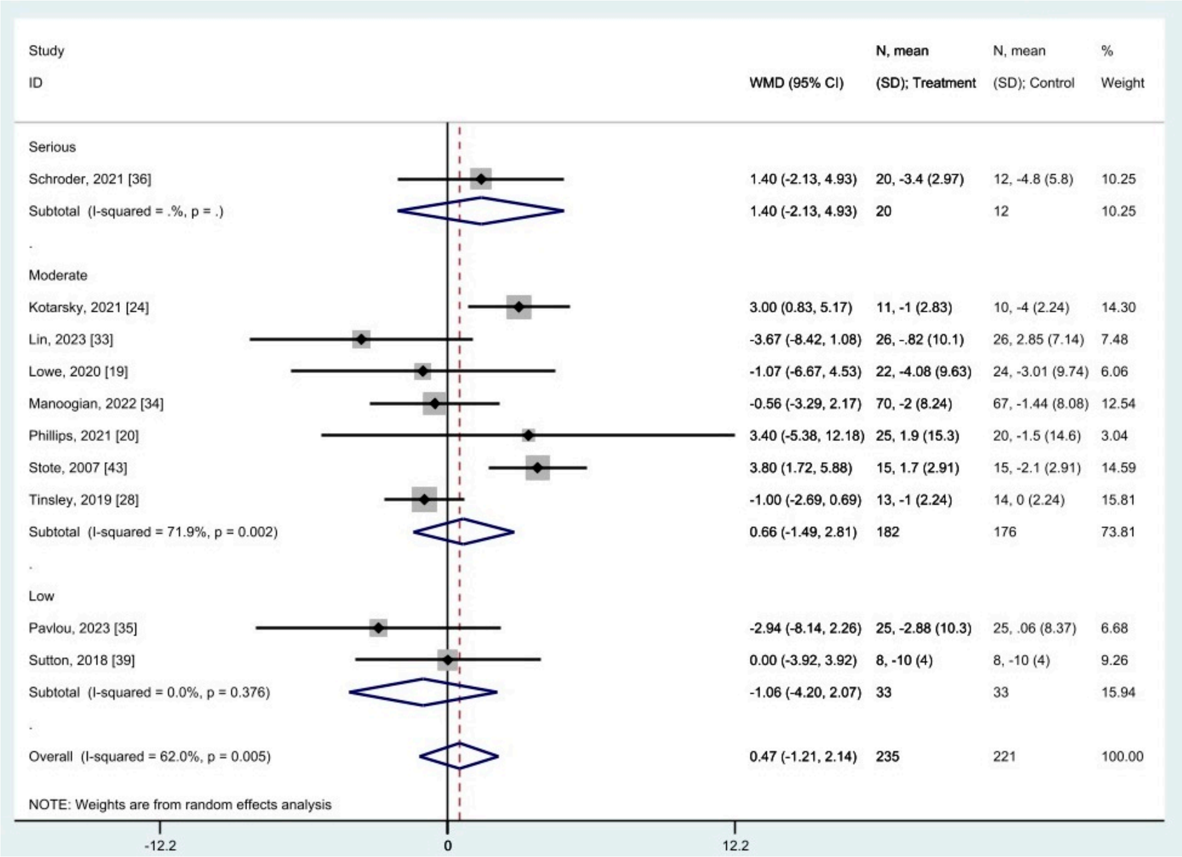

Supplemental Figure S12. Subgroup analysis for diastolic blood pressure by risk of bias

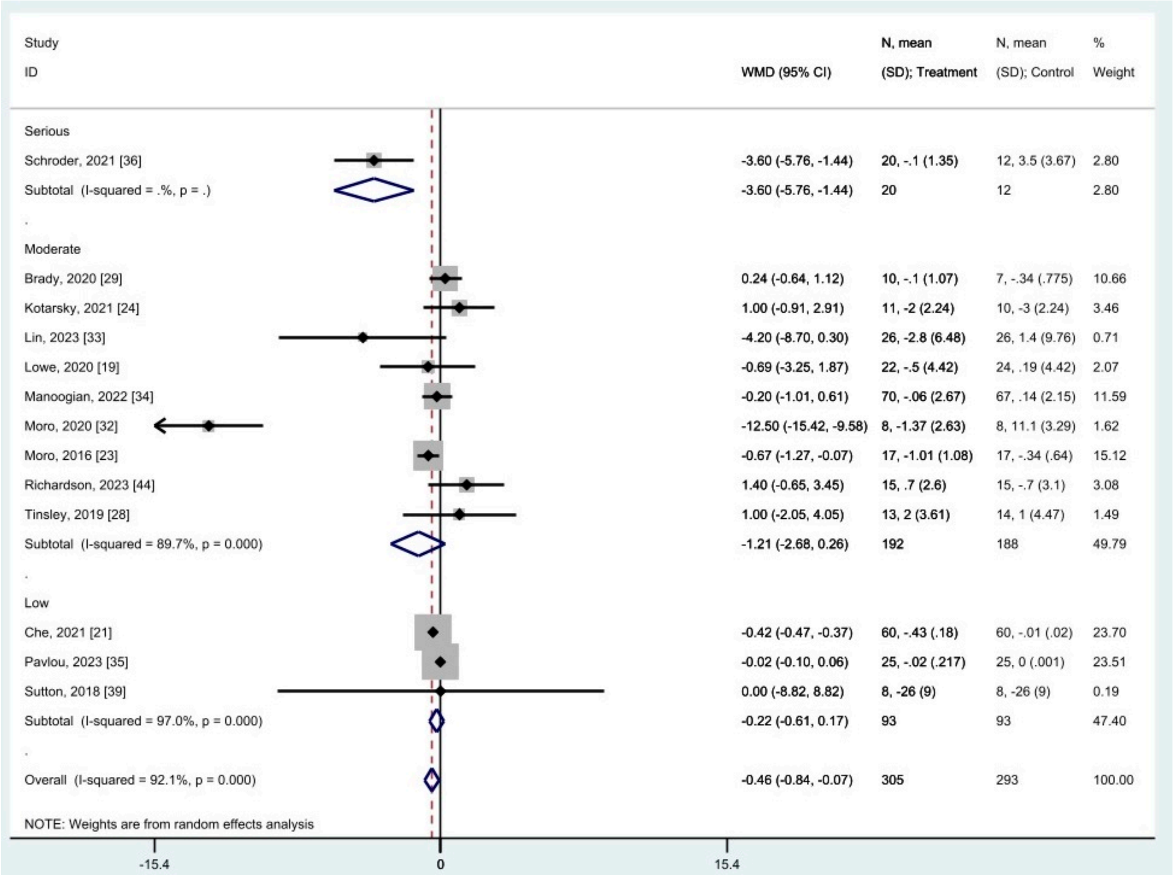

Supplemental Figure S13. Subgroup analysis for insulin by risk of bias

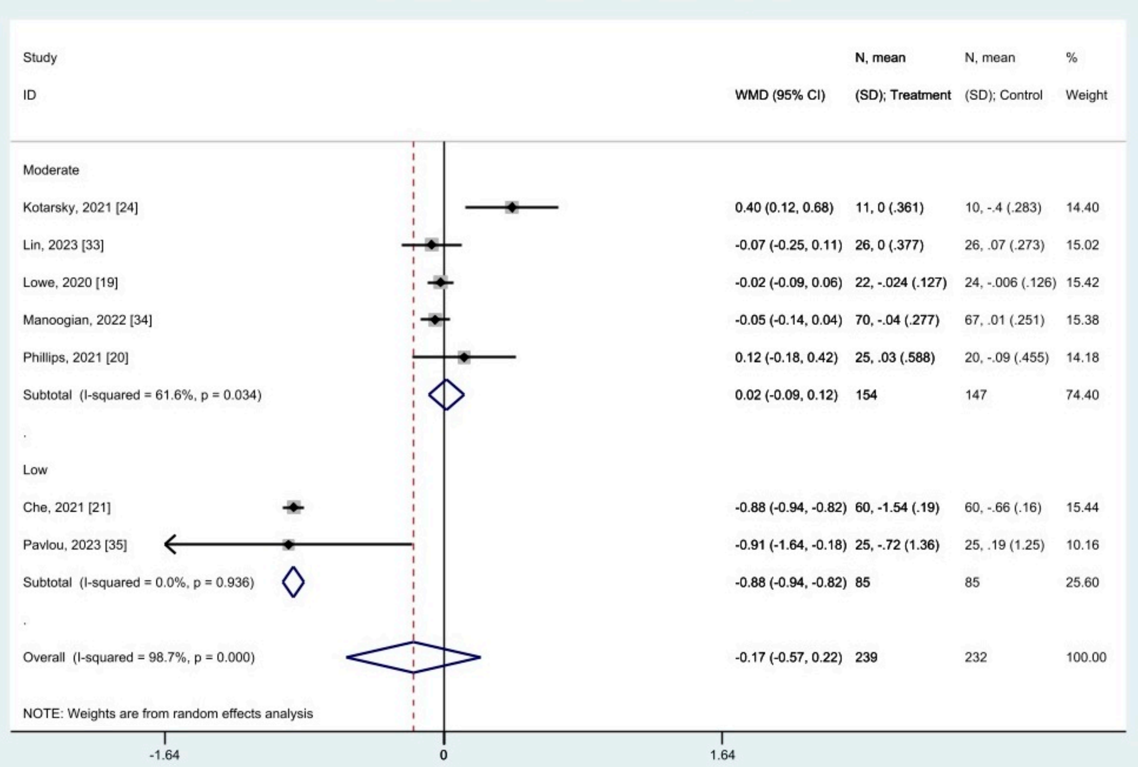

Supplemental Figure S14. Subgroup analysis for HbA1C by risk of bias

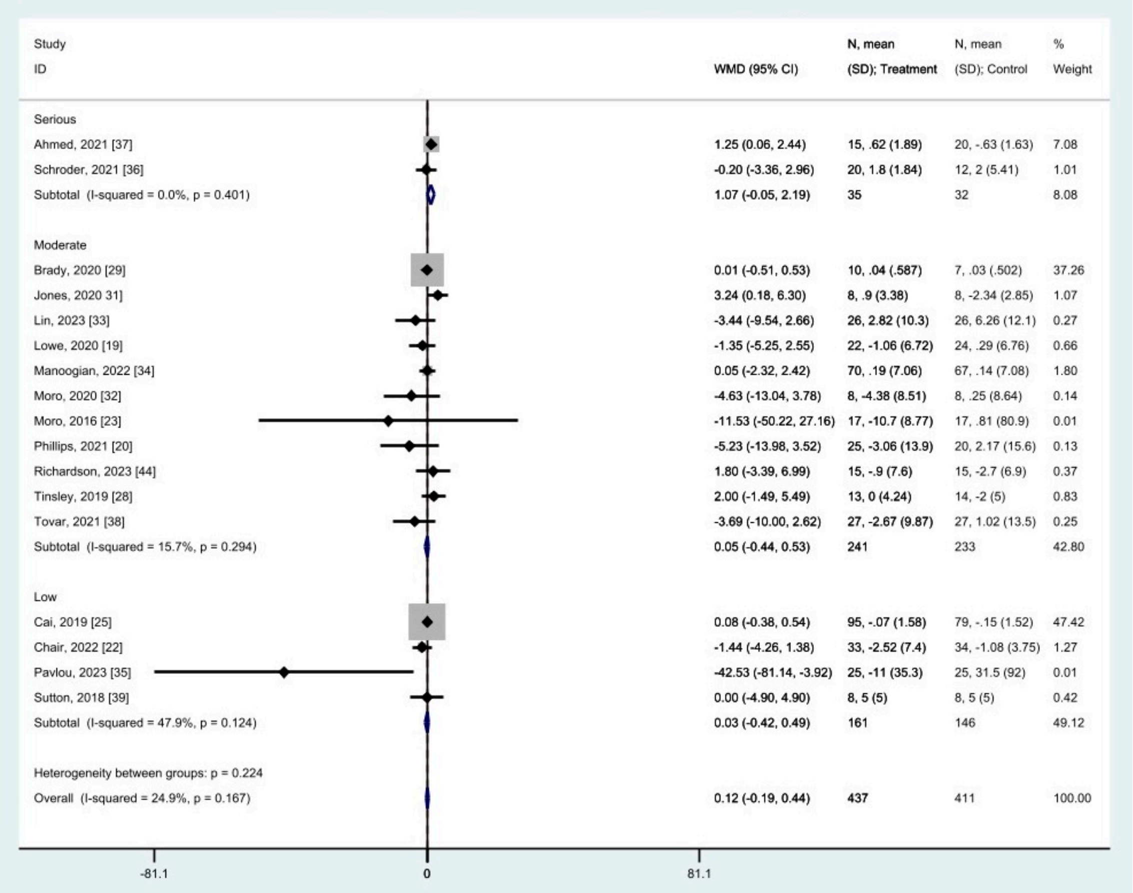

Supplemental Figure S15. Subgroup analysis for glucose by risk of bias

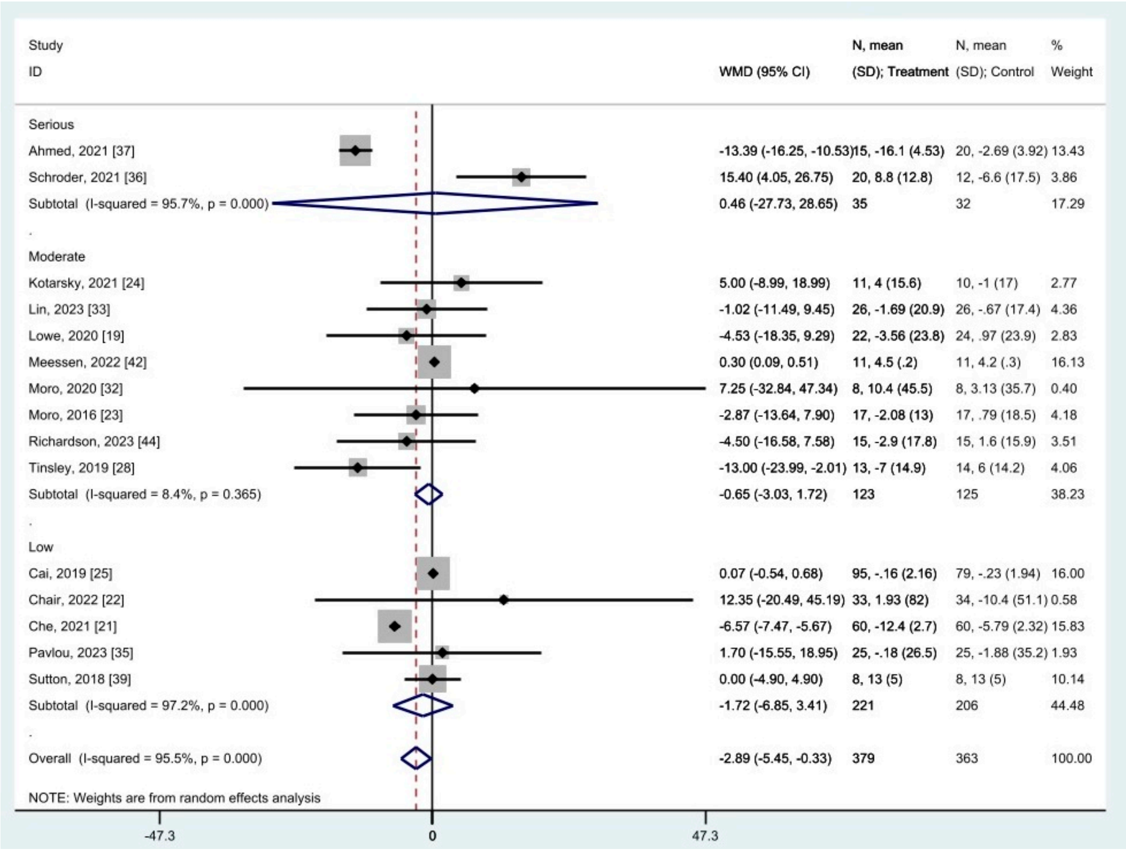

Supplemental Figure S16. Subgroup analysis for cholesterol by risk of bias

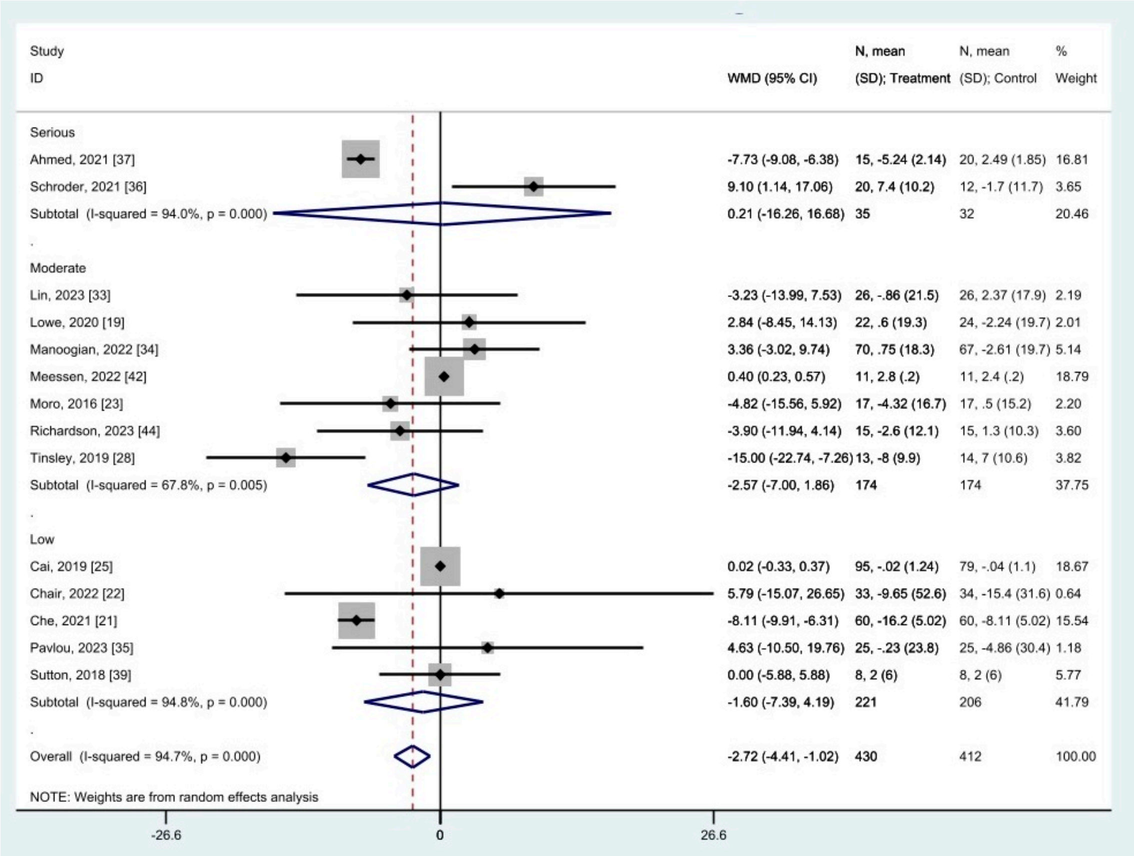

Supplemental Figure S17. Subgroup analysis for LDL by risk of bias

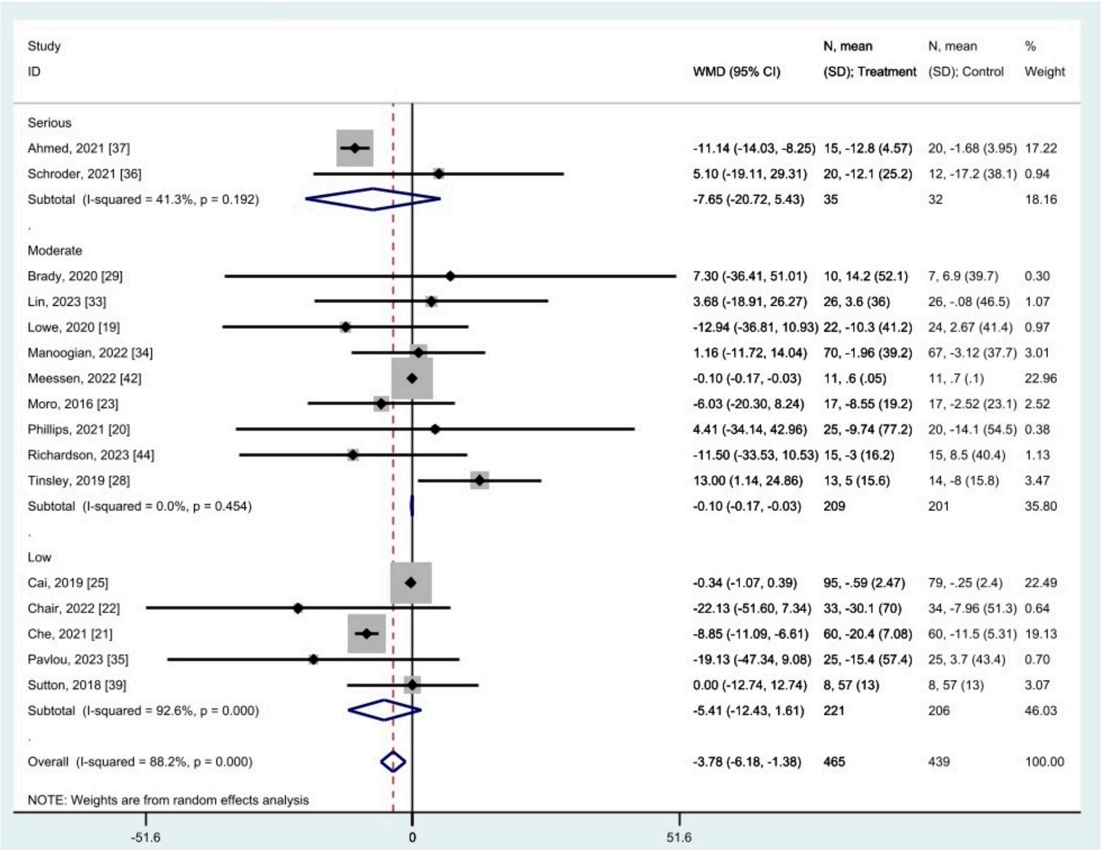

Supplemental Figure S18. Subgroup analysis for triglycerides by risk of bias

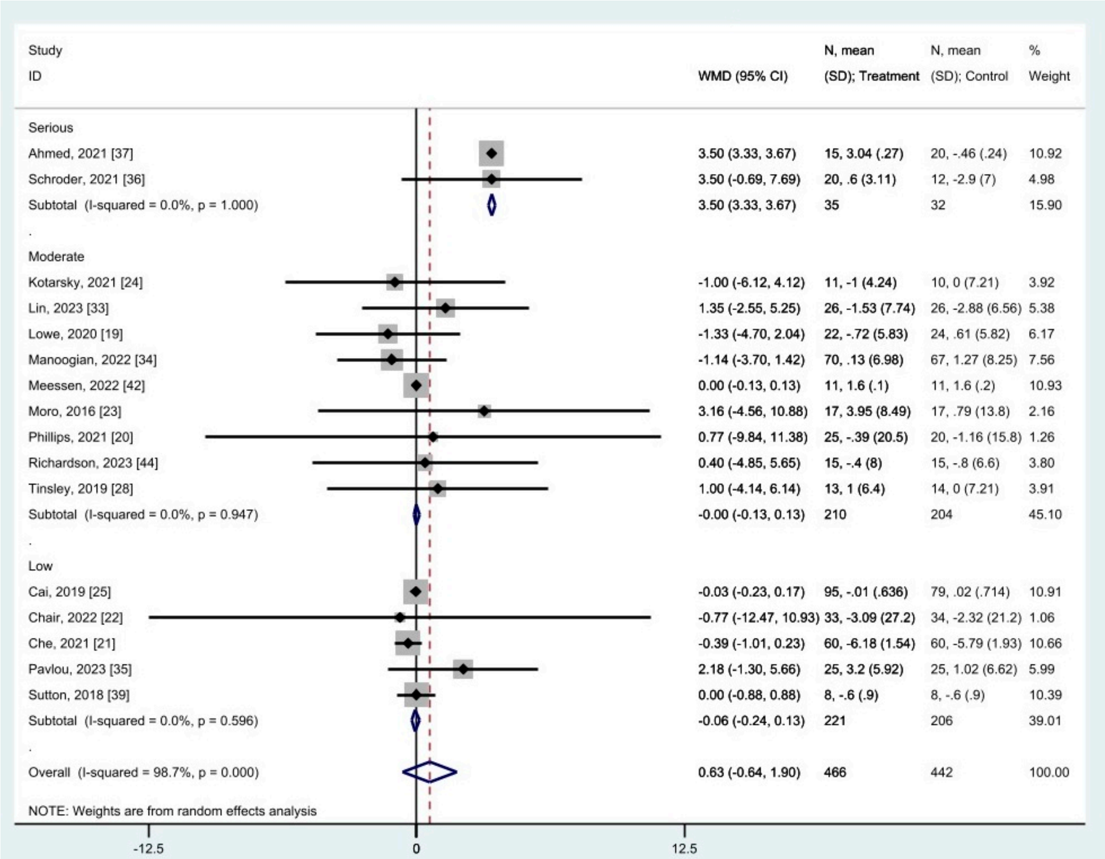

Supplemental Figure S19. Subgroup analysis for by risk of bias
