# Supplementary figures and images for: The Effect of Time-Restricted Eating on Cardiometabolic Risk Factors: A Systematic Review and Meta-Analysis
Source: Nutrients. 2024 Oct 30;16(21):3700. doi: 10.3390/nu16213700 (PMC11547938; doi:10.3390/nu16213700)

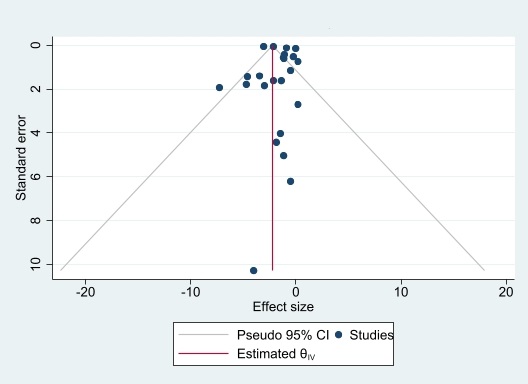

Supplement: Supplementary file 1 [file nutrients-16-03700-s001.zip › nutrients-3237280-Supplemental Figures-Funnel plots/Suppl.Fig. S1a. funnel plot for body weight.jpg]

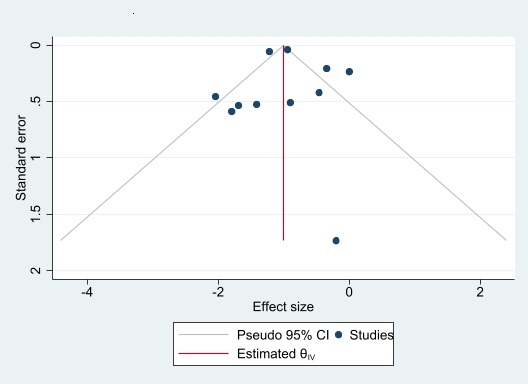

Supplement: Supplementary file 1 [file nutrients-16-03700-s001.zip › nutrients-3237280-Supplemental Figures-Funnel plots/Suppl.Fig. S1b. funnel plot for BMI.jpg]

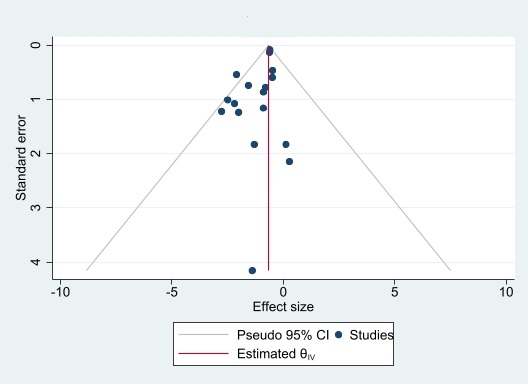

Supplement: Supplementary file 1 [file nutrients-16-03700-s001.zip › nutrients-3237280-Supplemental Figures-Funnel plots/Suppl.Fig. S1c. funnel plot for whloe body fat mass.jpg]

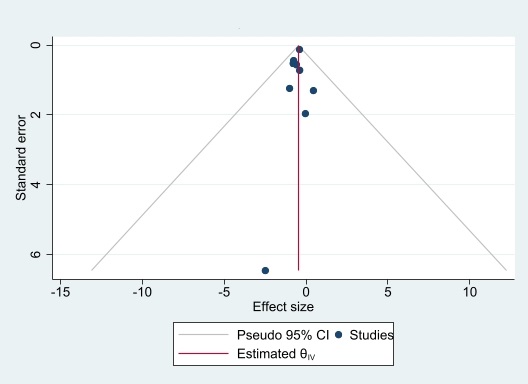

Supplement: Supplementary file 1 [file nutrients-16-03700-s001.zip › nutrients-3237280-Supplemental Figures-Funnel plots/Suppl.Fig. S1d. funnel plot for lean mass.jpg]

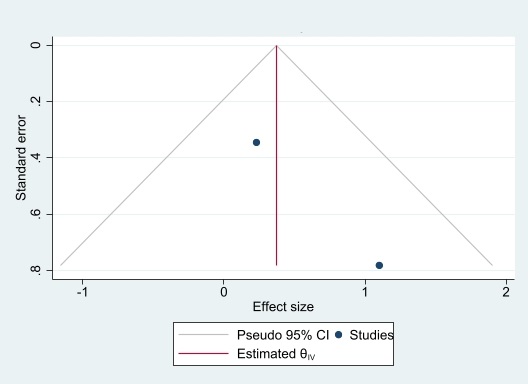

Supplement: Supplementary file 1 [file nutrients-16-03700-s001.zip › nutrients-3237280-Supplemental Figures-Funnel plots/Suppl.Fig. S1e. funnel plot for total body water.jpg]

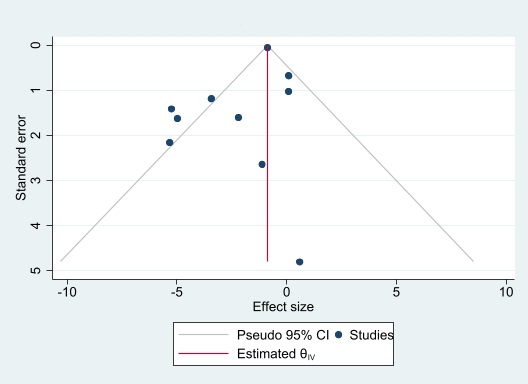

Supplement: Supplementary file 1 [file nutrients-16-03700-s001.zip › nutrients-3237280-Supplemental Figures-Funnel plots/Suppl.Fig. S2a. funnel plot for waist circumference.jpg]

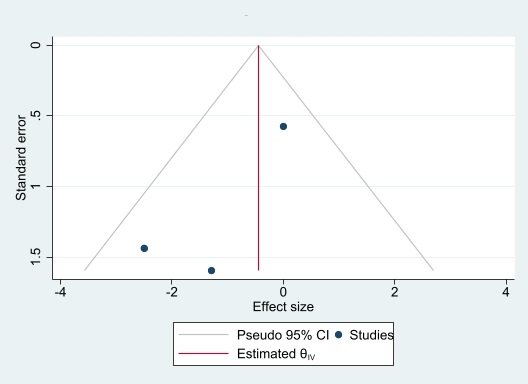

Supplement: Supplementary file 1 [file nutrients-16-03700-s001.zip › nutrients-3237280-Supplemental Figures-Funnel plots/Suppl.Fig. S2b. funnel plot for hip circumference.jpg]

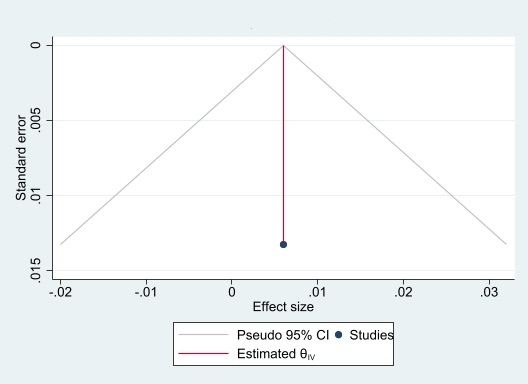

Supplement: Supplementary file 1 [file nutrients-16-03700-s001.zip › nutrients-3237280-Supplemental Figures-Funnel plots/Suppl.Fig. S2c. funnel plot for waist-to-hip ratio.jpg]

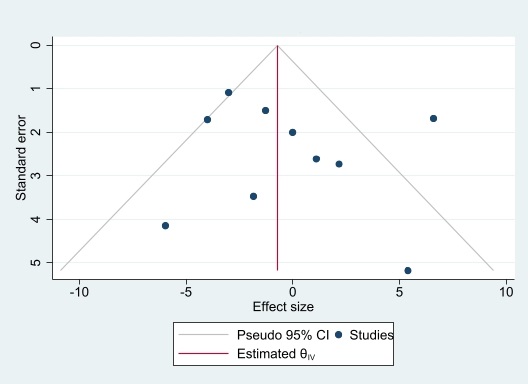

Supplement: Supplementary file 1 [file nutrients-16-03700-s001.zip › nutrients-3237280-Supplemental Figures-Funnel plots/Suppl.Fig. S3a. funnel plot for SBP.jpg]

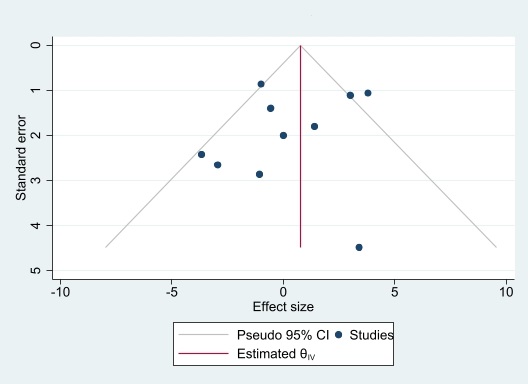

Supplement: Supplementary file 1 [file nutrients-16-03700-s001.zip › nutrients-3237280-Supplemental Figures-Funnel plots/Suppl.Fig. S3b. funnel plot for DBP.jpg]

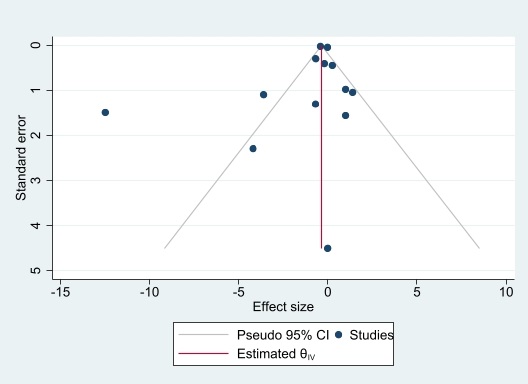

Supplement: Supplementary file 1 [file nutrients-16-03700-s001.zip › nutrients-3237280-Supplemental Figures-Funnel plots/Suppl.Fig. S4a. funnel plot for insulin.jpg]

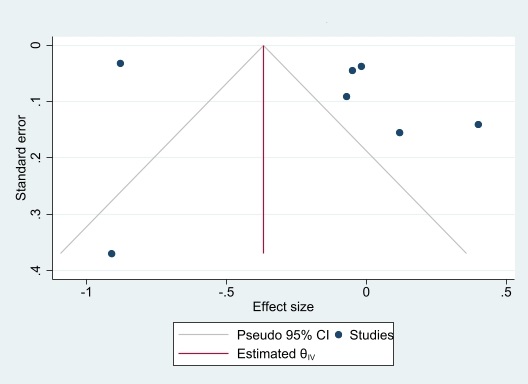

Supplement: Supplementary file 1 [file nutrients-16-03700-s001.zip › nutrients-3237280-Supplemental Figures-Funnel plots/Suppl.Fig. S4b. funnel plot for HbA1C.jpg]

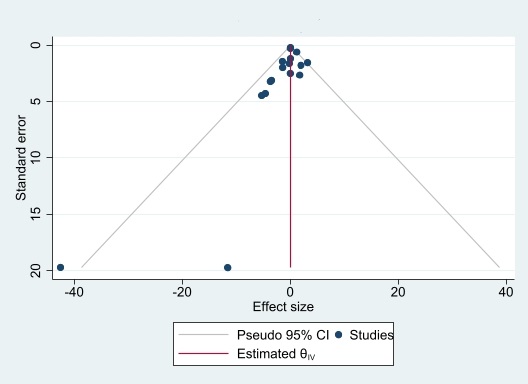

Supplement: Supplementary file 1 [file nutrients-16-03700-s001.zip › nutrients-3237280-Supplemental Figures-Funnel plots/Suppl.Fig. S4c. funnel plot for glucose.jpg]

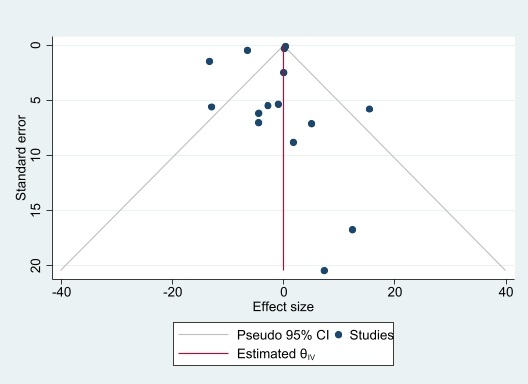

Supplement: Supplementary file 1 [file nutrients-16-03700-s001.zip › nutrients-3237280-Supplemental Figures-Funnel plots/Suppl.Fig. S5a. funnel plot for total cholesterol.jpg]

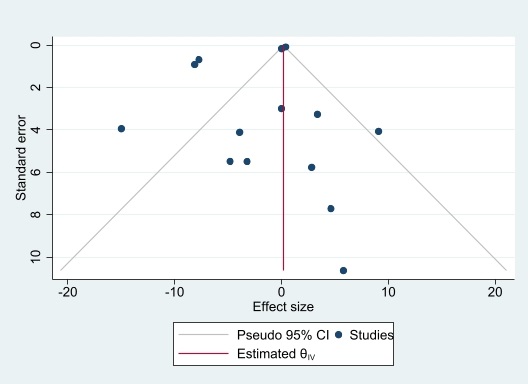

Supplement: Supplementary file 1 [file nutrients-16-03700-s001.zip › nutrients-3237280-Supplemental Figures-Funnel plots/Suppl.Fig. S5b. funnel plot for LDL.jpg]

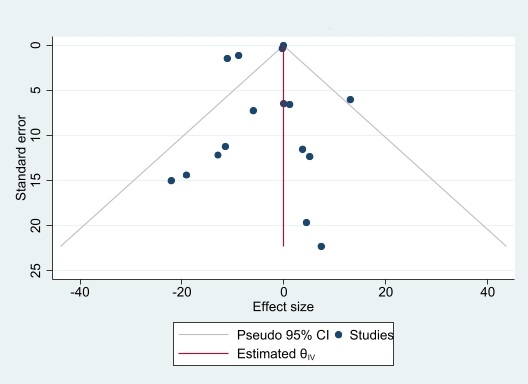

Supplement: Supplementary file 1 [file nutrients-16-03700-s001.zip › nutrients-3237280-Supplemental Figures-Funnel plots/Suppl.Fig. S5c. funnel plot for triglycerides.jpg]

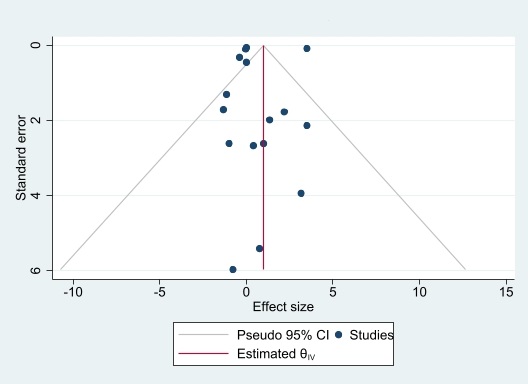

Supplement: Supplementary file 1 [file nutrients-16-03700-s001.zip › nutrients-3237280-Supplemental Figures-Funnel plots/Suppl.Fig. S5d. funnel plot for HDL.jpg]

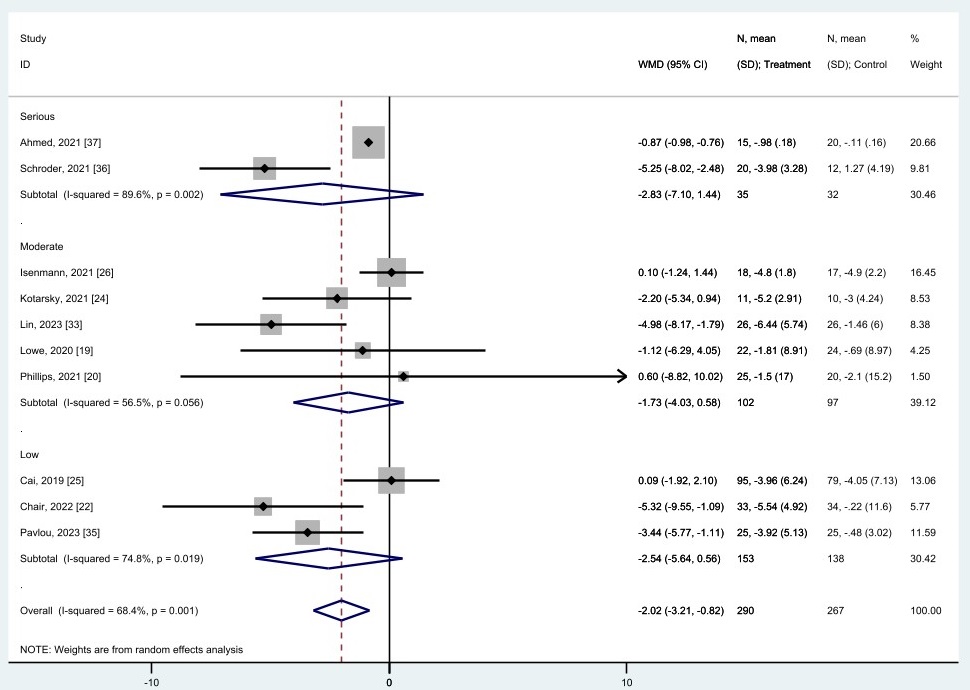

Supplement: Supplementary file 1 [file nutrients-16-03700-s001.zip › nutrients-3237280-Supplemental Figures-Subgroup Analysis/Suppl.Fig. S10. Subgroup analysis for waist circumference.jpg]

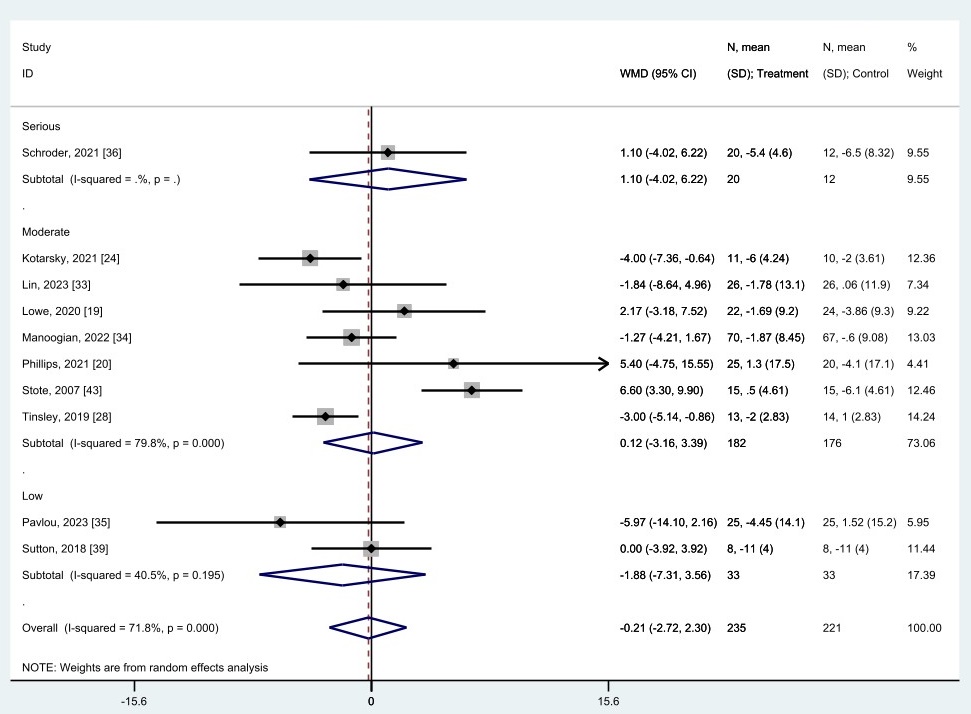

Supplement: Supplementary file 1 [file nutrients-16-03700-s001.zip › nutrients-3237280-Supplemental Figures-Subgroup Analysis/Suppl.Fig. S11. Subgroup analysis for SBP.jpg]

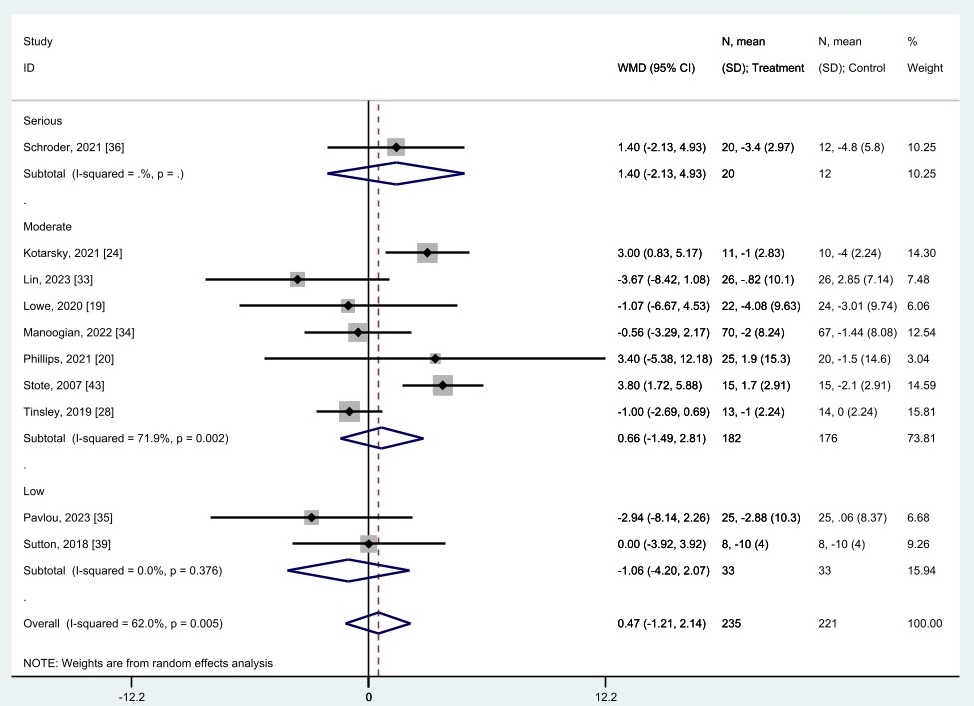

Supplement: Supplementary file 1 [file nutrients-16-03700-s001.zip › nutrients-3237280-Supplemental Figures-Subgroup Analysis/Suppl.Fig. S12. Subgroup analysis for DBP.jpg]

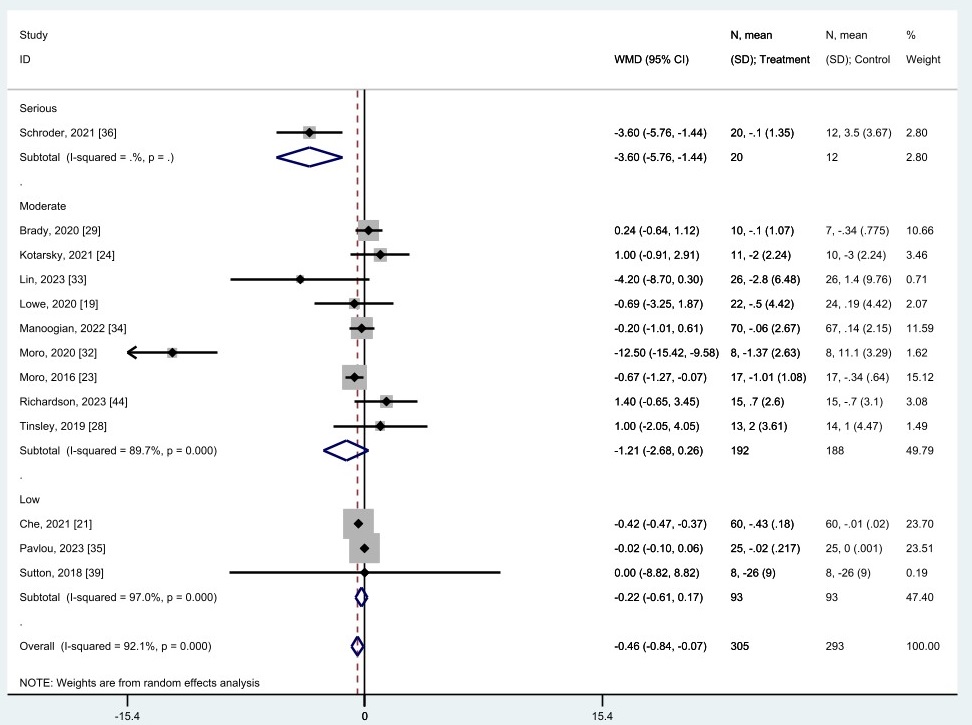

Supplement: Supplementary file 1 [file nutrients-16-03700-s001.zip › nutrients-3237280-Supplemental Figures-Subgroup Analysis/Suppl.Fig. S13. Subgroup analysis for insulin.jpg]

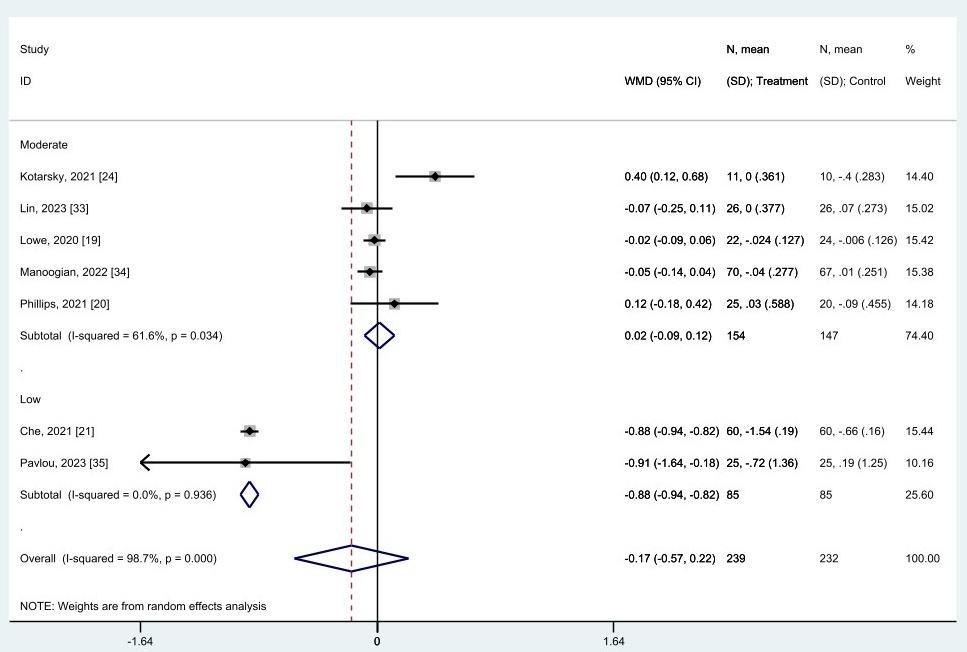

Supplement: Supplementary file 1 [file nutrients-16-03700-s001.zip › nutrients-3237280-Supplemental Figures-Subgroup Analysis/Suppl.Fig. S14. Subgroup analysis for HbA1C.jpg]

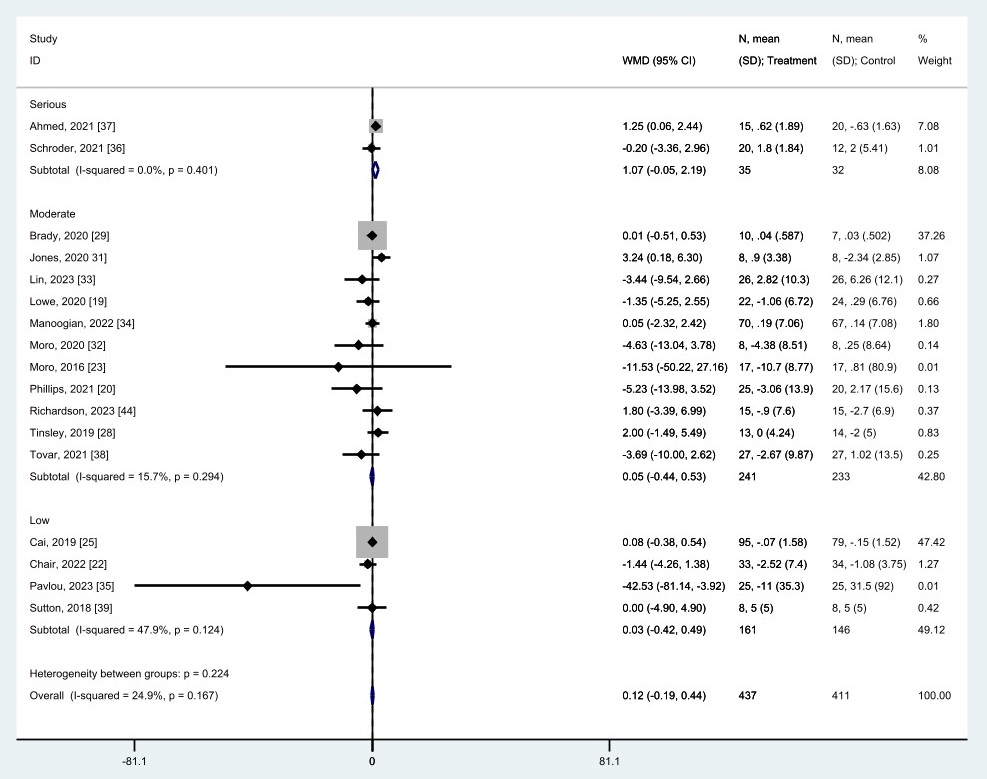

Supplement: Supplementary file 1 [file nutrients-16-03700-s001.zip › nutrients-3237280-Supplemental Figures-Subgroup Analysis/Suppl.Fig. S15. Subgroup analysis for glucose.jpg]

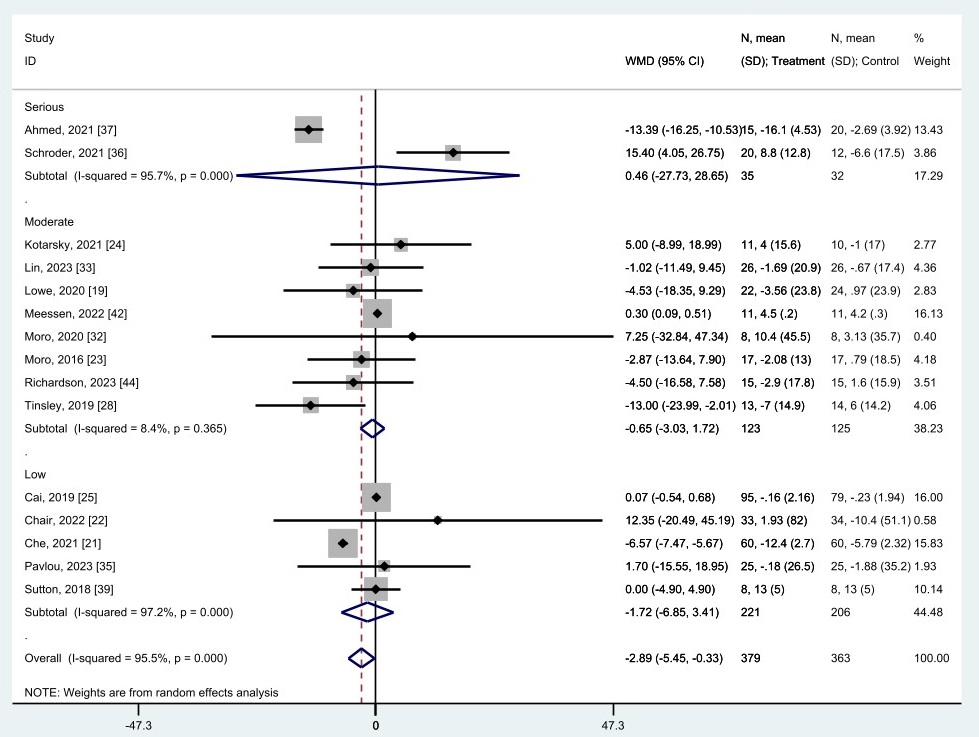

Supplement: Supplementary file 1 [file nutrients-16-03700-s001.zip › nutrients-3237280-Supplemental Figures-Subgroup Analysis/Suppl.Fig. S16. Subgroup analysis for cholesterol.jpg]

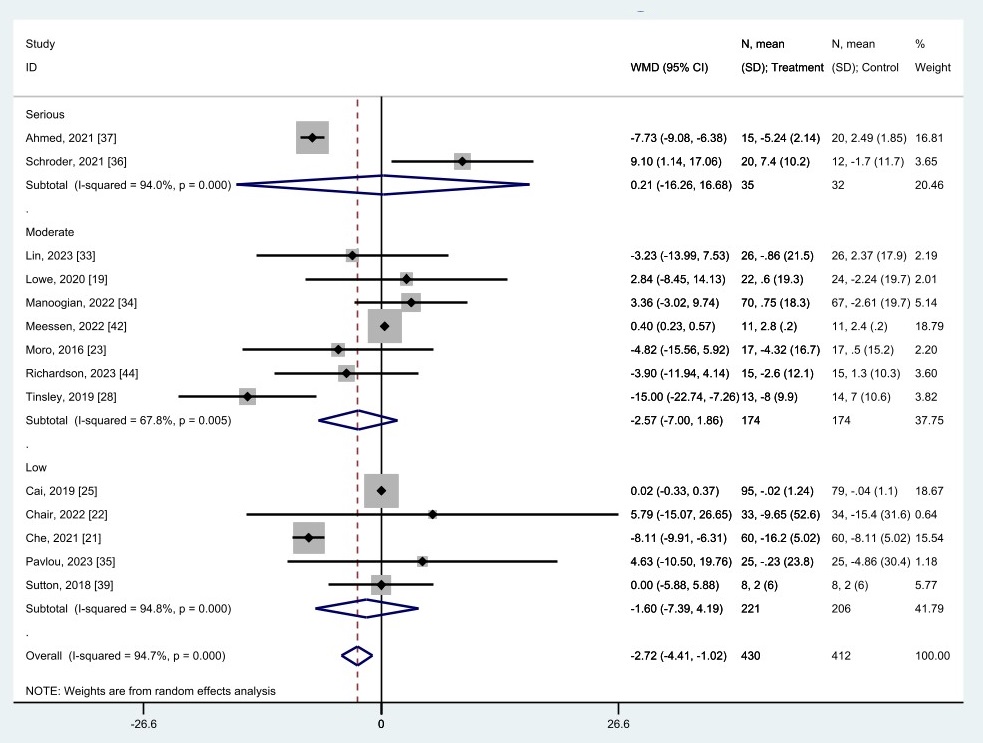

Supplement: Supplementary file 1 [file nutrients-16-03700-s001.zip › nutrients-3237280-Supplemental Figures-Subgroup Analysis/Suppl.Fig. S17. Subgroup analysis for LDL.jpg]

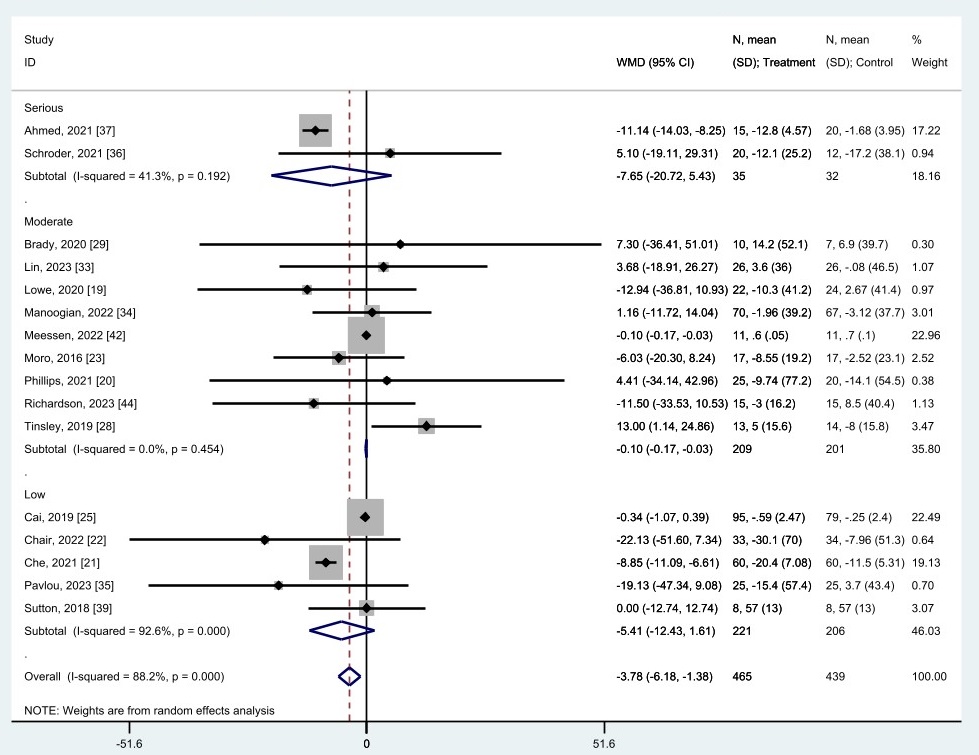

Supplement: Supplementary file 1 [file nutrients-16-03700-s001.zip › nutrients-3237280-Supplemental Figures-Subgroup Analysis/Suppl.Fig. S18. Subgroup analysis for triglycerides.jpg]

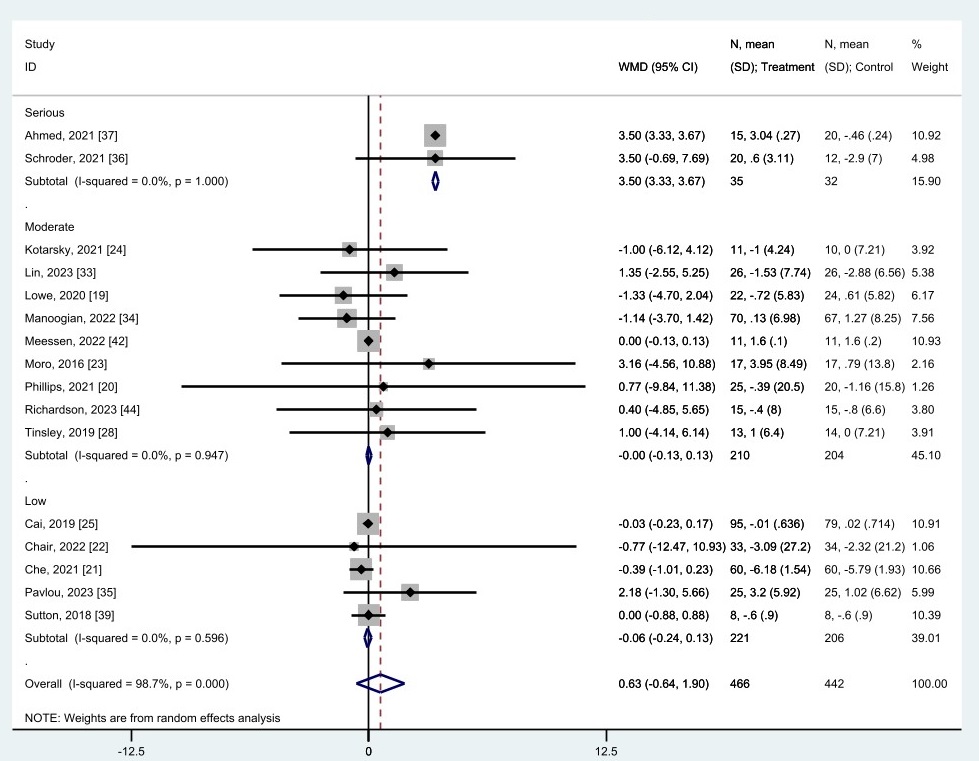

Supplement: Supplementary file 1 [file nutrients-16-03700-s001.zip › nutrients-3237280-Supplemental Figures-Subgroup Analysis/Suppl.Fig. S19. Subgroup analysis for HDL.jpg]

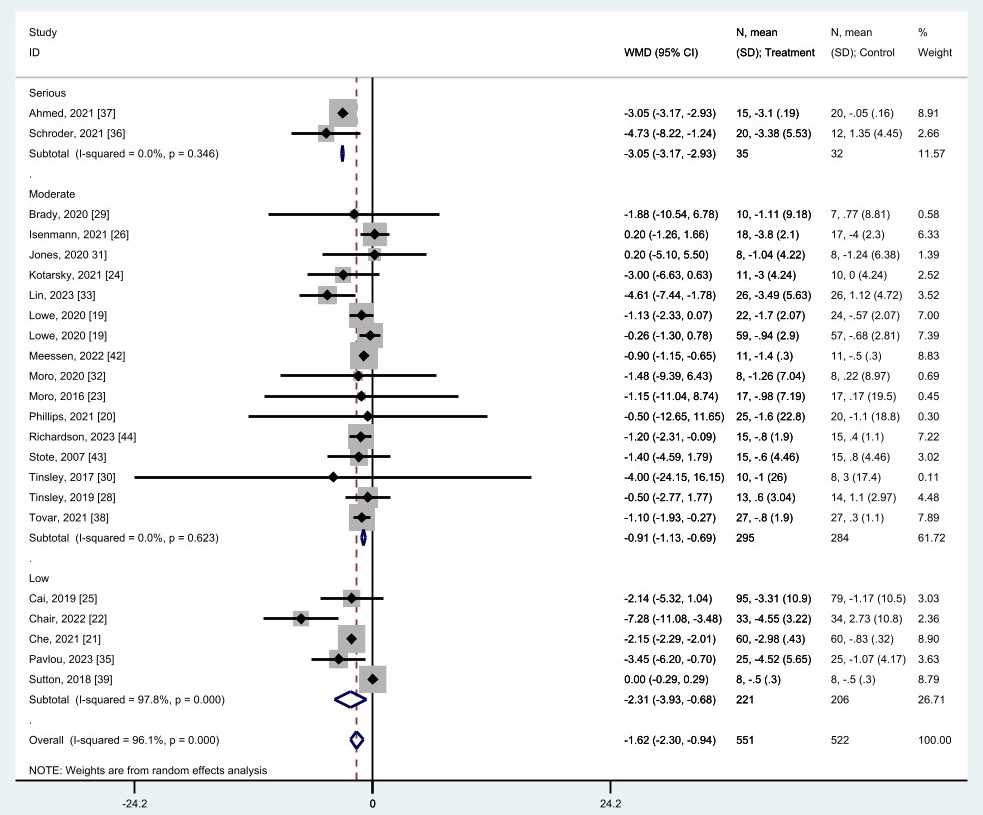

Supplement: Supplementary file 1 [file nutrients-16-03700-s001.zip › nutrients-3237280-Supplemental Figures-Subgroup Analysis/Suppl.Fig. S6. Subgroup analysis for body weight.jpg]

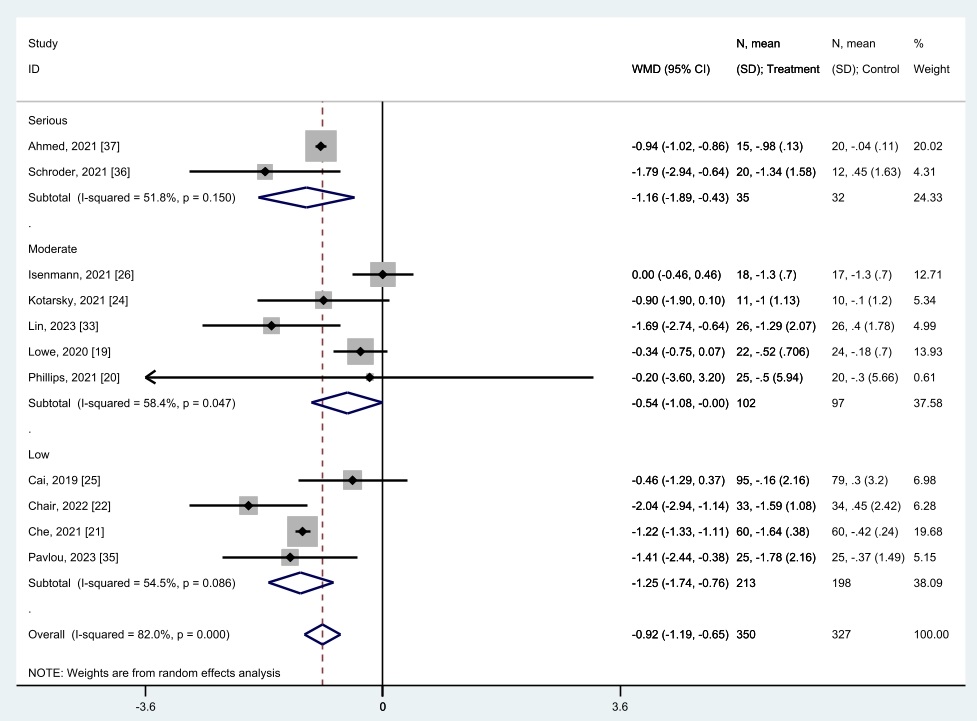

Supplement: Supplementary file 1 [file nutrients-16-03700-s001.zip › nutrients-3237280-Supplemental Figures-Subgroup Analysis/Suppl.Fig. S7. Subgroup analysis for BMI.jpg]

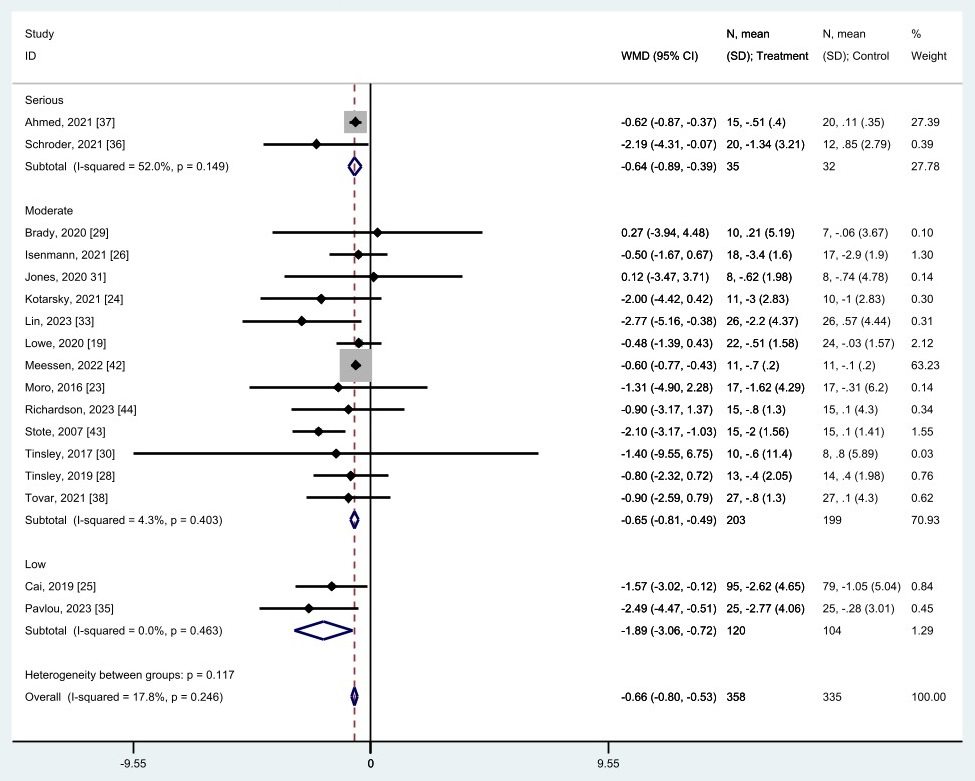

Supplement: Supplementary file 1 [file nutrients-16-03700-s001.zip › nutrients-3237280-Supplemental Figures-Subgroup Analysis/Suppl.Fig. S8. Subgroup analysis for whole body fat mass.jpg]

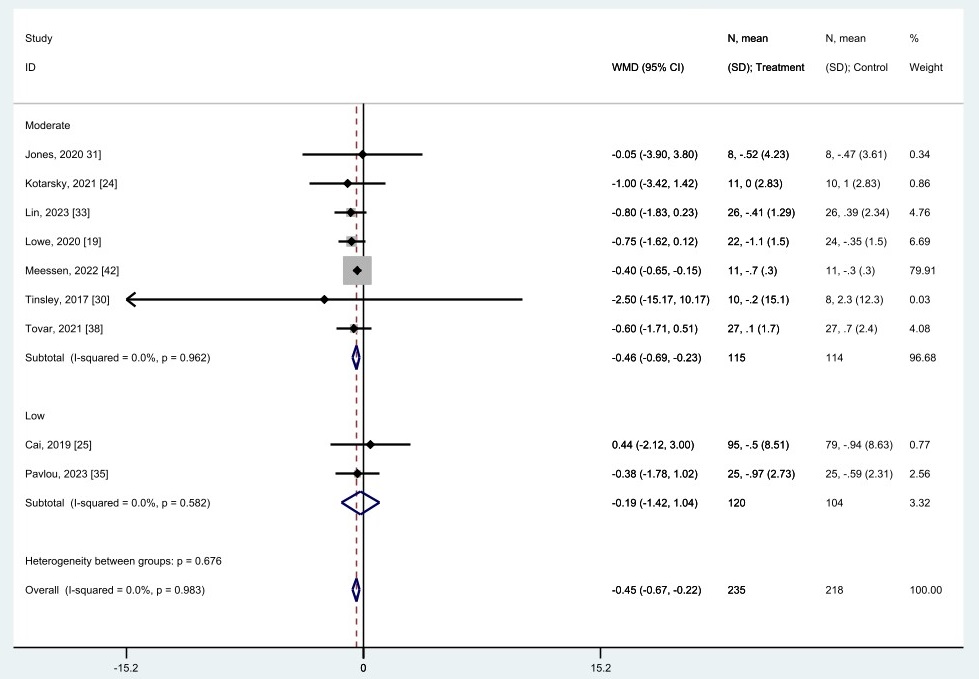

Supplement: Supplementary file 1 [file nutrients-16-03700-s001.zip › nutrients-3237280-Supplemental Figures-Subgroup Analysis/Suppl.Fig. S9. Subgroup analysis for lean mass.jpg]
